# Cell-Based Multi-Parametric Model of Cleft Progression during Submandibular Salivary Gland Branching Morphogenesis

**DOI:** 10.1371/journal.pcbi.1003319

**Published:** 2013-11-21

**Authors:** Shayoni Ray, Daniel Yuan, Nimit Dhulekar, Basak Oztan, Bülent Yener, Melinda Larsen

**Affiliations:** 1 Department of Biological Sciences, University at Albany, State University of New York, Albany, New York, United States of America; 2 Department of Computer Science, Rensselaer Polytechnic Institute, Troy, New York, United States of America; University of California San Diego, United States of America

## Abstract

Cleft formation during submandibular salivary gland branching morphogenesis is the critical step initiating the growth and development of the complex adult organ. Previous experimental studies indicated requirements for several epithelial cellular processes, such as proliferation, migration, cell-cell adhesion, cell-extracellular matrix (matrix) adhesion, and cellular contraction in cleft formation; however, the relative contribution of each of these processes is not fully understood since it is not possible to experimentally manipulate each factor independently. We present here a comprehensive analysis of several cellular parameters regulating cleft progression during branching morphogenesis in the epithelial tissue of an early embryonic salivary gland at a local scale using an on lattice Monte-Carlo simulation model, the Glazier-Graner-Hogeweg model. We utilized measurements from time-lapse images of mouse submandibular gland organ explants to construct a temporally and spatially relevant cell-based 2D model. Our model simulates the effect of cellular proliferation, actomyosin contractility, cell-cell and cell-matrix adhesions on cleft progression, and it was used to test specific hypotheses regarding the function of these parameters in branching morphogenesis. We use innovative features capturing several aspects of cleft morphology and quantitatively analyze clefts formed during functional modification of the cellular parameters. Our simulations predict that a low epithelial mitosis rate and moderate level of actomyosin contractility in the cleft cells promote cleft progression. Raising or lowering levels of contractility and mitosis rate resulted in non-progressive clefts. We also show that lowered cell-cell adhesion in the cleft region and increased cleft cell-matrix adhesions are required for cleft progression. Using a classifier-based analysis, the relative importance of these four contributing cellular factors for effective cleft progression was determined as follows: cleft cell contractility, cleft region cell-cell adhesion strength, epithelial cell mitosis rate, and cell-matrix adhesion strength.

## Author Summary

Branching morphogenesis is a complex and dynamic embryonic process that creates the structure of many adult organs, including the salivary gland. During this process, many cellular changes occur in the epithelial cells, including changes in cell-cell adhesions, cell-extracellular matrix (matrix) adhesions, cell proliferation, and cellular contraction, resulting in formation of clefts in the epithelial cells of the organ. A comprehensive understanding of the relative contributions of these cellular processes has crucial therapeutic implications for organ regeneration and functional restoration of organ structure in diseased salivary glands. Here, we have developed a cell-based model of cleft progression and simulated cleft progression under conditions of altered cell-cell adhesions, cellular contractility, cell-matrix adhesion and cell proliferation to identify the optimum cellular conditions that cause clefts to progress. The model predicts that cleft progression requires a moderate level of cleft cell contractility, a low epithelial proliferation rate, reduced cell-cell adhesion strength in the cleft and high cell-matrix adhesion strength also in the cleft region. The results of our classification analysis demonstrate that cellular contractility in the cleft cells has a significant effect on cleft progression, followed by cell-cell adhesion strength, rate of cell proliferation, and strength of cell-matrix adhesion energies.

## Introduction

Branching morphogenesis is a specific type of tissue morphogenesis that is a crucial developmental process occurring in several organs, such as the mammary glands, lungs, kidney, and salivary glands to maximize epithelial surface area for secretion or absorption of fluids and gases [1]. The process of branching morphogenesis is complex and dynamic, requiring reciprocal interactions between the epithelium and the mesenchymal cell types [2], [3]. Since many organs develop by branching morphogenesis, one strategy for a regenerative medicine-based restoration of diseased or damaged branched organs would be to reactivate the cellular and molecular mechanisms that produce these organs during development. Deciphering the coordinated mechanisms driving branching morphogenesis is therefore relevant to the basic understanding of development and may be applicable to future regenerative medicine strategies.

Submandibular salivary gland (SMG) is one of the best-characterized organ systems for the study of branching morphogenesis [4] since the embryonic organs can be grown ex vivo and manipulated genetically [5] or pharmacologically [6]–[9] and monitored using time-lapse imaging [10], [11]. The gland starts to develop at embryonic day 11 (E11) when the epithelium protrudes into the neural crest-derived mesenchyme. At E12, clefts, or indentations, initiate in the surface of the primary epithelial bud, which progress inward towards the interior of the epithelium, subdividing the primary bud into multiple buds by E13. Cleft progression is associated with proliferation of the epithelial cells causing tissue outgrowth [2]. In successive days, embryonic development continues into postnatal development with continued cleft formation and bud outgrowth together with duct formation, thereby forming a highly arborized adult structure. Cellular differentiation begins at E15, concomitant with continued branching to create functional cell types, leading to saliva secretion [3]. Since the salivary glandular structure is presumably important to facilitate its function, the question of how this ramified epithelial structure is established has been the subject of many biological studies and some recent computational modeling studies.

Analysis of the physics of complex systems has demonstrated that collective behaviors arising from ensembles of a large number of interacting components cannot be interpreted from behavioral analysis of individual components [12]. Thus, several researchers have utilized various systems biology and computational modeling approaches as tools to try and understand salivary gland morphogenesis [13]. Starting at the organ level, Lubkin's group developed a 2D model for cleft formation during early salivary gland branching morphogenesis. In this work, the epithelium and mesenchyme were both modeled as immiscible Stokes fluids, separated by an interface representing the basal lamina. Using a 2D model, they predicted that mesenchymal viscosity drives a clefting force that affects the time required for branching and that the ratio of viscosities of the epithelium to mesenchyme affects the shape of clefts [14]. In subsequent work, they developed a more complex 3D model that incorporated the mesenchyme-generated traction forces. This model predicted that these mesenchymal traction forces were sufficient to drive cleft formation [15]. Although these computational models were the first attempt in modeling complex tissue-driven forces and were able to successfully generate clefts, the cleft shape did not mimic the actual shape observed in the developing salivary glands. Additionally, the 3D model could not explain how branching morphogenesis can occur in the absence of mesenchymal cells when epithelial rudiments are grown in an artificial basement membrane together with growth factors [10], [16]–[19]. The fact that branching morphogenesis can occur without mesenchymal cells indicates that a cell-based model system that focuses on epithelial cellular processes may have utility in modeling the process of cleft formation.

Previous experimental research using ex vivo embryonic organ explants and transgenic mouse models has made possible the identification of many molecules and cellular processes required for cleft formation in the submandibular salivary gland; however an integrated model for cleft formation does not exist. Using a cell-based modeling environment we set out to incorporate as much of the experimental data as possible into a computational model. Early work indicated that actin microfilaments are required for forming clefts [20], [21]. Since actin is known to regulate cell shape, a simple model for cleft formation was proposed where localized actin contraction at the basal cell surfaces alternating with contraction at the apical surfaces in the outer monolayer of epithelial cells bends this peripheral cell layer to generate clefts. However, subsequent electron microscopy studies did not detect basal actin bundles [11]. According to recent experimental work, cleft formation can be subdivided into four fundamental steps: initiation, stabilization, progression and termination. While the events leading to cleft initiation remain unclear, recent studies indicate that cleft stabilization requires formation of cell-ECM adhesions containing active focal adhesion kinase (FAK) [7]. Initiated clefts can only progress when they have been stabilized by an inside-out integrin signaling that promotes activation of focal-adhesion protein complexes that can overcome a presumed mechanochemical barrier to progression. Cleft progression was shown to require Rho kinase I (ROCK I)-stimulated non-muscle (NM) myosin II/-mediated actomyosin contractility for basal fibronectin (FN) assembly in the cleft region and associated cell proliferation, at least part of which is stimulated by FN [6]. FN assembly induced epithelial cell proliferation, which had a major impact on cleft progression and bud outgrowth but not on cleft initiation. Explants treated with hydroxyurea, a known pharmacological S-phase inhibitor, showed a reduction of progressive clefts with no effect on number of initiated clefts as compared to vehicle control glands [6]. With time-lapse imaging studies, Kadoya and Yamashina [11] showed that clefts progress with a very subtle replacement of cell-cell adhesions with cell-ECM adhesions with very little space between the cells on each side of the cleft. They proposed that local folding of the plasma membrane near the base of the cleft produces a “shelf” containing an accumulation of actin filaments. The shelf was proposed to be the contact point between the epithelium and matrix, and the cleft progressed in the groove between the shelf and the cleft cell walls, through retraction of the groove [11]. Cleft formation was also found to be accompanied by accumulation of FN in the cleft bases and concomitant loss of adjacent E-cadherin based cell-cell junctions [5]. This conversion of cell-cell adhesions to cell-matrix adhesions was found to be regulated transcriptionally through increases in BTB (POZ) domain containing 7 (Btbd7) to activate a local epithelial-to-mesenchymal transition (EMT) found near the base of the cleft [22]. Btbd7 is assumed to assist in separating the adjacent epithelial cells, while assembled FN keeps accumulating at the newly separated cleft base cells, promoting continuous cleft progression [23].These experimental studies point to a coordinated requirement for cell proliferation, actomyosin contractility, cell-cell adhesions and cell-matrix adhesions in cleft progression.

To develop a relevant cellular level model of morphodynamic pattern formation in developing salivary glands, we used a modeling environment that specifically attempts to simulate several cellular events including mitosis or cell proliferation, actomyosin contraction, cellular organization with cell-cell interactions and cell-matrix interactions and allows independent computational manipulation of each parameter within specific cell populations. The Glazier-Graner-Hogeweg (GGH) model [24], [25] was originally developed to model cellular rearrangements as a function of inter-cellular surface energy, cell membrane fluctuations and energy between cells and their external environment [26]. The GGH model has been utilized to recapitulate cellular events during pattern formation and morphogenetic movements in several organisms and organ systems [27]–[31]. The GGH model represents each cell as an aggregation of lattice points, or pixels, in a 2D space. Each cell is assigned an energy signature denoting the probability of the cell to grow, move, adhere, and organize into different patterns. GGH thus enables cell-centered modeling to simulate changes in collective ensembles of cells within tissues to facilitate the testing of how specific cell behaviors affect a larger morphological process.

In this study, we construct a GGH model of salivary gland cleft progression using CompuCell3D (CC3D), an open-source implementation of the GGH model. We developed both a single cleft and a whole epithelial tissue model, which include GGH-based representations of cellular adhesions, cellular contractility, cell-matrix adhesions and cell proliferation within the epithelial cells that are surrounded by a simplified mesenchymal compartment. The whole tissue model demonstrated a mutual dependence of cleft progression on neighboring clefts, and the single cleft model was used to investigate the contribution of the cellular parameters to individual cleft progression. We used morphometric quantification of cleft depths from time-lapse images of ex-vivo cultured glands to create a temporally and spatially accurate model. The clefts obtained during the simulations were assessed for quality using three morphometric features – cleft depth, cleft spanning angle, and cleft tilt angle. Comparisons with ex-vivo cultured glands were generated from image data that was measured using the same features. Using the single cleft model we have been able to make the following predictions regarding the contributions of cellular parameters to branching morphogenesis: (i) cleft progression requires an intermediate level of actomyosin contractility in the cleft region, and lower contractility is more detrimental to cleft progression than higher levels of contractility, (ii) proliferation rates and location of the proliferating cells affect cleft progression such that very low proliferation rates are required and an equal number or majority of the proliferating cells should be in the outer columnar epithelial layer rather than in the inner cells, (iii) low levels of cell-cell adhesion in the cleft promote progressing clefts, and (iv) cell-matrix adhesions do not have as significant an effect on cleft progression as do cell-cell adhesions. Since it is difficult to make assessments of the relative importance of cellular factors to branching morphogenesis using experimental methods, we used ex-vivo data-sets to formulate three classes of cleft progression and used classifiers to identify the most important factors during cleft progression. Our results show that epithelial cell contractility in the cleft cells is the most influential factor during cleft progression, closely followed by mitosis rate and cell contractility.

## Material and Methods

### Ethics Statement

This study involves CD1 mice and was approved by the University at Albany, SUNY IACUC under protocol numbers 09-013 and 12-013.

### The Glazier-Graner-Hogeweg (GGH) Model

The GGH model is built on the energy minimization-based Ising model, using imposed fluctuations via a Monte Carlo approach [24]. The simulation space is divided into a lattice, which may be two- or three-dimensional, and cells are represented by groups of adjacent lattice points; each lattice point has an associated energy value that is assigned based on its interactions with other lattice points. Energy is also assigned to cells based on cell-cell interactions, and the sum of energies across all lattice points and cells in the simulation space is the effective energy. The energy assignment of a lattice point is based on functions representing biological behaviors or constraints, and the effective energy of the simulation can be written as a Hamiltonian equation, where each term represents the sum contribution of a particular energy function. The model is based on the assumption that the most favorable state is the lowest energy state.

To develop the 2D GGH single cleft and tissue models of cleft progression, we used the following terms:

Contact energy represents differential adhesion between model cells of different types by assigning an energy penalty to adjacent lattice points belonging to different cells. Each possible pair of cell types (τ_a_,τ_b_) is enumerated and assigned an energy penalty J(τ_a_,τ_b_), including same-type pairs. Cell types that adhere to each other are assigned a lower energy penalty; the cell type τ of a particular lattice point i is given by τ_σ(i)_, where σ is the cell ID. The contact energy penalty assigned to a pair of lattice points (i,j) is therefore given as J(τ_σ(i)_,τ_σ(j)_). To prevent lattice points within the same cell from being assigned a contact energy penalty, this is multiplied by (1−δ_σ(i)_,_σ(j)_), where δ is the Kronecker delta. The term for contact energy in the Hamiltonian equation across all pairs of lattice points (i,j) is therefore given as:
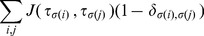
(1)for all neighboring lattice sites i, j.

Area (a) represents cell volume in two dimensions. It is a cell-based energy function that penalizes cells for deviating from a target size, simulating the biological tendency for cells to grow to and maintain a certain size. It has two constants, a target area A, and a strength factor λ. The term is thus:
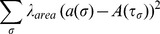
(2)for each cell σ and cell type τ

Perimeter (p) is a representation of surface area in two dimensions. Like area, it is a cell-based energy function, and it imposes an additional constraint on cell size based on the amount of plasma membrane available to a cell. It also uses two constants, a target perimeter P, and a strength factor λ. The energy term is given as:
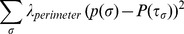
(3)for each cell σ and cell type τ

Focal point plasticity is a cell-based energy term that assigns an energy penalty for linked cells that deviate from a target length L, based on the distance between the cell centroids (l). Although it was developed to simulate actomyosin-dependent contractility, it is used in our model to simulate the effects of actomyosin contractility-dependent FN assembly. Since we are unable to represent the FN wedge as a physical structure, we reproduce its cleft-forming effects by exerting a separating effect on opposing cells of the cleft wall through FPP. Within the cleft, the target distances between opposing cells are assigned based on depth, and represent the shape constraint imposed by the FN structure. The λ value modulates the effect of focal point plasticity, and corresponds to the amount of actomyosin contractility present in the simulation. The energy term is:
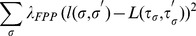
(4)for linked cells σ and σ', and cell type τ

The target distances that produce the characteristic shape of the cleft are assigned based on an inverse relationship with the depth; cells near the bottom of a cleft are assigned shorter target distances than the cells at the top of the cleft. This relationship was determined through examination of images of progressed clefts from time-lapse images of embryonic day 12 (E12) organ explants. Additionally, we used a simplified two-cell model to investigate the effects of FPP relative to Cell-Matrix (CM) contact energy, λ, and target distance, for constant cell-cell contact energy (CC) value = 10 to determine the values of λ to use in the model (Figure S4).

The full Hamiltonian equation for our simulation is thus given as the sum of these four equations:
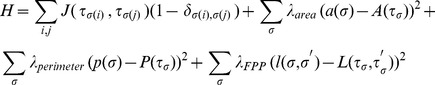
(5)


Energy minimization is carried out by choosing pairs of adjacent lattice points from different cells, and an attempt is made to copy the cell ID from the first point to the second. This copy attempt grows one cell, either by forcing another cell to shrink, or expanding into the medium. The effective energy is calculated before and after the change, and if the new energy is lower, the change is made permanent. However, if the resulting energy is higher, the change is only retained with some probability using a Boltzmann acceptance function, e^−ΔH/T^. In the context of the GGH simulation, T is a constant that controls the intrinsic motility of the cell, corresponding to the amplitude of cytoskeletally derived membrane fluctuations. Using T, we have allowed a certain amount of cell motility. Allowing some amount of energy-raising lattice-copy events is important as it prevents the model from stalling at local energy minima. A single step in the GGH model actually consists of N lattice copy attempts, where N is the total number of lattice sites in the simulation space. These attempts are carried out through a Monte Carlo simulation using modified Metropolis dynamics, designated as Monte Carlo steps (MCS) [24].

Cell proliferation in the GGH model is accomplished by dividing an existing cell into two equally sized new cells. To simulate mitotic cells, a subset of cells is instructed to grow to twice their original size and divide every 100 Monte Carlo steps (MCS), mimicking the growth and mitosis of biological cells.

Simplification: Although the GGH model is able to mimic parameters such as growth factor absorption kinetics, we have omitted these from this initial study to reduce complexity and focus on the cellular behaviors. Similarly, we have simplified the basement membrane and mesenchymal compartment, which contains nerves and blood vessels [2], [32] in addition to mesenchymal fibroblasts; surrounding the epithelium into a single compartment we call “matrix” and that is often called “medium” in GGH models. The matrix compartment is essentially represented here as a single special GGH cell that is not subjected to area and perimeter constraints. We have not included apoptosis in our model since there is currently no biological data to suggest that apoptosis is important in cleft progression.

### Ex-vivo Submandibular Salivary Gland Organ Culture

Embryos from timed-pregnant female mice (strain CD-1, Charles River Laboratories) at embryonic day 12 (E12) (with day of plug discovery designated as E0), were used to obtain submandibular salivary gland rudiments (SMGs) following protocols approved by the University at Albany, SUNY IACUC committee (protocols 09–013 and 12-013), as reported previously [6], [7], [33], [34]. E12 SMGs that contain 1 primary bud were micro-dissected from mandible slices and cultured, as described previously. For culturing ex-vivo organs, 13 mm, 0.1 µm pore size Nucleopore Track-Etch membrane filters (Whatman) were used. The SMGs were floated on top of the filters that sit on 200 µL of 1∶1 DMEM/Ham's F12 Medium (F12) lacking phenol red (Invitrogen) in glass-bottomed 50 mm microwell dishes (MatTek Corporation). The medium was supplemented with 50 µg/mL transferrin, 150 µg/mL L-ascorbic acid, 100 U/mL penicillin, and 100 µg/mL streptomycin, to make complete DMEM/F12 medium. Brightfield images were acquired on a Nikon Eclipse TS100 microscope equipped with a Canon EOS 450D digital camera at 4X (Plan 4X/0.10 NA) magnification.

### Whole-Mount Immunocytochemistry and Confocal Imaging

Whole-mount immunocytochemistry was performed as previously described [6], [7], [33], [34]. E12 SMGs were fixed in 4% paraformaldehye (PFA) in 1X phosphate buffered saline (1XPBS) containing 5% (w/v) sucrose for 20 min at room temperature. SYBR Green I (1∶10000, Invitrogen) was used to detect nuclei and proliferating cells were detected using phospho-Histone H3 (pHH3) antibody (1∶100, Cell Signaling Technology). Epithelium was detected using an antibody recognizing E-cadherin (1∶250, BD Biosciences), F-actin was detected using Alexa Fluor 546 Phalloidin (Invitrogen, 1∶350), and mesenchyme was detected using an antibody recognizing PDGF receptor (R)-β (1∶100, Epitomics). Appropriate cyanine dye-conjugated AffiniPureF (ab′)2 fragments were used as secondary antibodies (Jackson ImmunoResearch Laboratories, 1∶100). SMGs were imaged on a Zeiss LSM510 confocal microscope at 20X (Plan Apo/0.75 NA), or 63X (Plan Apo/1.4 NA) magnification.

### Confocal Time-Lapse Series Acquisition

E12 SMG organ explants were treated with 200 µl of Hank's balanced salt solution (HBSS lacking Ca^2+^ or Mg^2+^, Life Technologies) containing 0.4% (v/v) dispase (Life Technologies) for 25 min at 37°C, and the mesenchyme was physically removed by microdissection, as described in [10]. The epithelial rudiment was cultured in a final concentration of 6 mg/mL Matrigel (BD Biosciences) diluted in DMEM/F12 containing 20 ng/mL EGF and 200 ng/mL FGF7 (R&D Systems). The gland was imaged using time-lapse microscopy with a 20X objective lens using a Zeiss 510 Meta Confocal microscope. 120 images were captured as 5 µm sections at 10 minute intervals for a 20 hour time period using the MultiTime macro. The 543 nm laser was used to capture a near-DIC image. Images were captured at a 512×512 pixel resolution using a scan speed of 9 in line averaging mode. A total of 30 glands were imaged for 20 hours in three separate sets and 40 clefts were measured using image analysis software ImageJ [35]. The first frame and the last frame (after 20 hours) were used to measure the depth in pixels for each cleft and according to the scale, the distances were converted to micrometers (µm).

### Image Processing

To enhance the contrast of the grey-scale pHH3 images and the SYBR green images, we applied the contrast-limited adaptive histogram equalization algorithm (CLAHE) [36] to the image. The CLAHE algorithm considers the image as a collection of smaller regions and applies histogram equalization on these regions. The objective of histogram equalization is to transform the image so that the intensity histogram of the output image approximately matches a specified histogram; in our case we use a curved histogram. The CLAHE algorithm evens out the distribution of used grey values and thus makes hidden features of the image more visible. Noisy regions of the images are removed by considering regions of intensity greater than a pre-determined threshold. For the E-cadherin marker images, we applied a Gaussian smoothing followed by the CLAHE algorithm, and then removed noisy regions based on a predetermined threshold. Binary masks were created for the SYBR green and pHH3 histogram equalized images by applying an OR operation on the histogram equalized image and the E-cadherin marker. The total area of the connected components in both images was calculated, and the ratio yielded the percentage of SYBR green-positive cells (total cells) that are in mitosis, or M phase, of the cell cycle.

### Classification Analysis

Four values for mitosis rate (MR), six values for contractility (FPP λ), five values for cleft region adhesion (CC), and five values for cleft-matrix adhesion (CM) were chosen from the hypothesis driven individual analyses, and 40 simulations were run for each of the 600 possible combinations. Cleft simulations were classified as failed (less than 17.8 µm), non-progressive (17.8 to 30.5 µm), progressive (30.5 to 40.7 µm), and super-progressive (greater than 40.7 µm) based on minimum, first quartile, and third quartile depths of ex-vivo cleft measurements. Parameter combinations were assigned an overall class based on the cleft depths attained in a majority class within the 40 runs; in the case of a tie, the median depth was used to classify the parameter combination. This resulted in 275 failed, 188 non-progressives, 85 progressive, and 52 super-progressive results. To determine the importance of each GGH parameter in cleft progression, we formulated the problem as a supervised learning feature selection task, with each combination as a data point and the parameter values as features. Samples were created by drawing 50 random points from each class. For each of the 15 possible combinations of the four features, a 10-fold cross-validation using a radial basis kernel support vector machine (SVM) was performed on the sample, reporting the training and testing accuracies [37]. A greater decrease in classification accuracy corresponds to a more important feature. Additionally, analysis of the parameters resulting in progressive clefts was performed to confirm the importance of each parameter; parameters that were essential to progressive clefts were expected to be distributed around a particular value with low variance.

## Results

### Establishing a Single-Cleft GGH Model for SMG Branching Morphogenesis

**Figure 1:**
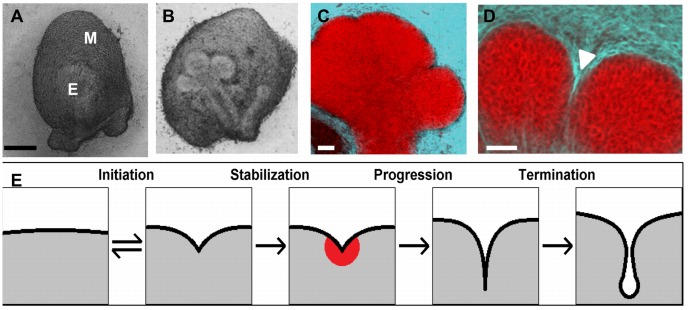
Mouse submandibular salivary gland organ structure and cleft formation during branching morphogenesis. Brightfield images of (a) an embryonic day 12 (E12) submandibular salivary gland (SMG) organ explant and (b) an SMG explant harvested at E12 and grown for 24 hours ex vivo with epithelium (E) and mesenchyme (M) labeled. Scale, 200 µm. Single confocal images of E12 SMGs following ICC to detect epithelium (E-cadherin, red) and mesenchyme (PDFGR, cyan) captured at (c) cleft initiation and (d) a late stage of cleft progression. Progressing clefts are indicated (white arrow head). Scale = 50 µm. (e) Diagram depicting cleft transitions.

We chose to start our model at E12, when the mouse SMG undergoes the first round of branching morphogenesis. At E12, the gland is a single epithelial mass, or bud, atop a stalk, surrounded by a condensed mesenchyme (Figure 1A, 1B). Clefts initiate as indentations in the epithelium, which progressively furrow interiorly. Since cleft initiation and cleft progression are biochemically independent steps [6] and little biological information is available regarding mechanisms of cleft initiation, we chose to pre-specify an individual initiated cleft in the model and simulate only the stage of cleft stabilization and cleft progression (Figure 1E). At E12, the epithelium expresses E-cadherin (Figure 1C,1D) but later stage differentiation marker proteins are not yet expressed [38], [39]. We therefore assumed that the cell-cell adhesions present are E-cadherin-containing adherens junctions with an absence of tight junctions, as previously reported [38], [39]. The epithelium is surrounded by mesenchyme that expresses PDGFR-β, which can be used to distinguish the latter from the former (Figure 1C, 1D). Closely associated with the epithelial cells is the basement membrane, a specialized extracellular matrix (ECM) that forms a boundary between the epithelial and mesenchymal tissue compartments [5], [40]. Since we are focusing on epithelial cell parameters that control cleft progression, we modeled the basement membrane and the entire mesenchyme compartment as a simplified single cell, designated as “matrix,” which lacks area and perimeter constraints.

### Designating Epithelial Cell Subpopulations in the CC3D Modeling Environment

**Figure 2:**
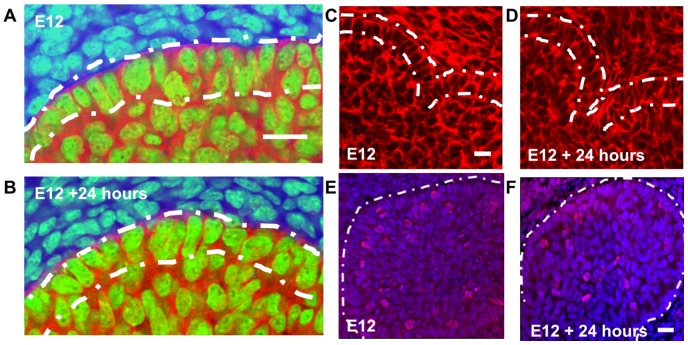
Cellular and cytoskeletal organization in developing salivary glands. Epithelial cells express E-cadherin (red) during organ development and organize as polarized outer columnar cells (OCCs) and non-polarized inner polymorphic cells (IPCs) at (a) E12 and (b) retain this organization after 24 hours of growth. Cortical F-actin localization occurs during cleft formation at (c) E12 and (d) E12+24 hrs. Epithelial proliferation occurs in both outer and inner cell compartments shown with phospho-histone H3-labeled nuclei (red) relative to total nuclei with SYBR green (blue) at (e) E12 and (f) E12+24 hrs. Scale = 20 µm.

**Figure 3:**
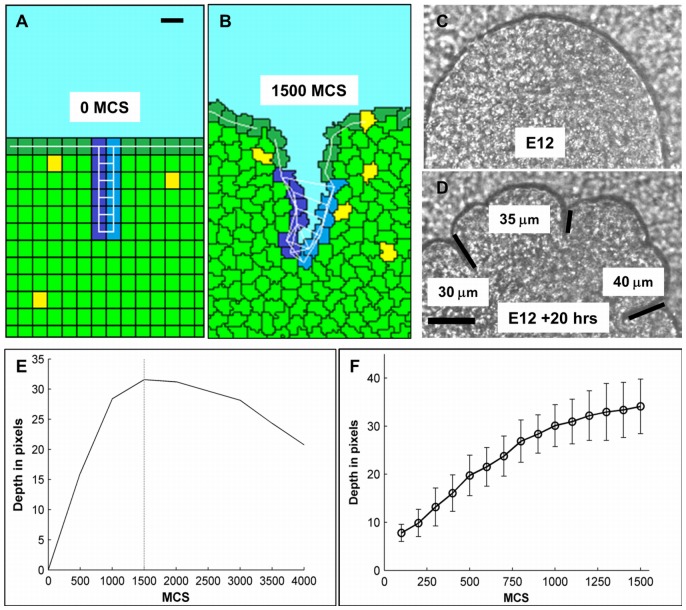
Construction of a GGH model of cleft formation and scope of modeling. A six cell deep single cleft was designed having 36 pixels as the total cleft depth with predefined cleft cells (dark and medium blue). The local cleft simulation shows the other epithelial areas as polarized OCCs (dark green) and non-polarized IPCs (light green) with mitotic cells (yellow). The mesenchymal compartment (cyan) has been simplified to a single large cell. FPP links in the OCCs are shown as white lines. Spatial conversion: 1 µm = 1.06 pixels. Temporal conversion: 1 MCS = 48 sec. Single cleft model at (a) 0 Monte Carlo steps (MCS) and (b) 1500 MCS (Scale = 50 µm). Time lapse images of a mesenchyme-free E12 epithelial rudiment at (c) time 0 hr and (d) time 20 hrs with cleft measurements under 200× magnification (Scale = 20 µm). Average cleft depth = 36.2 µm. (e) Since cleft depth reaches a maximum value at 1500 MCS, this value was selected to represent the end of cleft progression. (f) The cleft depth distribution over time for the base case condition showing 34.1 pixels cleft depth after 1500 MCS, corresponding to a 20 hr growth period of a pre-defined initiating cleft through the end of cleft progression.

At E12, there are two structurally distinct epithelial precursor cell populations [34], [38]. The outer columnar cells (OCCs) that contact the basement membrane surround a cluster of less organized inner polymorphic cells (IPCs) (Figure 2A), and this cell arrangement is maintained during 24 hours of ex-vivo culture (Figure 2B). The 6×6 pixel square cells were arranged in a homogenous grid, a simplification that approximates the initial cell distribution with OCCs labeled in dark green and IPCs in light green (Figures 3A, 3B). To calibrate the model with image data, we performed time-lapse imaging of multiple E12 mesenchyme-free SMG organ explants for 20 hours and measured the length of the resulting clefts (Figures 3C, 3D, Video S1). Clefts achieved an average depth of 36.2 µm and a median depth of 35 µm. Based on the cleft depths obtained from the time-lapse analysis, we defined normal cleft depth in the CC3D model as 36 pixels, using 6 cells per cleft, shown in light and deep blue (Figures 3A, 3B). To distinguish OCCs from IPCs, we use a baseline perimeter equivalent to the perimeter of a square for the initial cell area. Relative to this baseline, we allow a marginal increase in the target perimeter for IPCs, which encourages them to take on more irregular shapes, whereas OCCs were confined to a smaller perimeter, encouraging them to maintain a more ordered columnar shape as they do in-vivo.

### Designation of Adhesion Properties

Cells exhibit differential adhesion that can drive complex tissue-level behavior [30]. The IPCs demonstrated a slightly more diffuse distribution of the adherens junction protein E-cadherin than the OCCs, suggestive of reduced adherence of the IPCs to each other [38]. To represent cell-cell adhesions in the GGH model, we start with a baseline contact energy penalty; increasing or decreasing the penalty simulates lower and higher adhesion, respectively, as explained by Eq. 1. Relative to this baseline, we designated increased cell-cell contact energy between IPCs to represent decreased adhesion properties and decreased contact energy between OCCs, simulating a possible increased adhesion that may help OCCs maintain their regular shape. During cleft progression, contact energy between the OCCs representing the cleft walls is directed to increase relative to the baseline, while contact energy between cleft cells and the matrix is decreased. This decrease in contact energy allows cell-matrix contacts to be established between the cleft cells.

The basement membrane is a dynamic structure that plays a critical role in branching morphogenesis, and cell-matrix adhesions are known to change dynamically during branching morphogenesis [5], [6], [8], [10]. In the GGH model, we represent basement membrane through the contact energy settings between the OCCs and the matrix, which is represented as a single homogenous cell not subject to area and perimeter constraints. This contact energy is designated in our model as the “cell-matrix” contact energy and behaves as defined by Eq. 1.

### Designation of Cellular Contractility

The actin cytoskeleton has long been known to be required for branching morphogenesis and was specifically shown to be required to maintain initiated clefts [20], [21]. In salivary gland epithelial cells, the actin cytoskeleton is organized primarily into cortical actin filaments at the cell perimeter (Figure 2C, 2D) in an E12 organ explant grown ex vivo for 0 or 24 hours. Our subsequent work indicated that actin and non-muscle (NM) myosin II–mediated contraction are required to regulate cleft progression [6]. The current model for cleft progression assumes that actomyosin contraction is required for assembling fibronectin through integrin activation [5], [6], [7], which then stimulates local EMT through upregulation of Btbd7 and Slug and reduction of E-cadherin levels [22]. Since EMT is one of the chief factors promoting cleft progression, we utilized variable cell-cell and cell-matrix contact energies to facilitate cleft progression. Without any other energy factors affecting cleft progression, the resultant clefts were poorly formed (Video S2).

During early cleft formation, the cleft evolves as a thin opening between OCC cells, possibly primarily aided by random cell movements [5], [11] and possibly from a hypothesized force generated by FN assembly [10] pushing assembled basement membrane into the cleft opening. FN assembly, dependent on strength of actin contractility for integrin activation, might cause the two cleft-forming epithelial cell layers to separate. FN assembly also stimulates proliferation [6], presumably causing an outward force that emanates from inside the bud to counteract an inward cleft movement force produced by FN.

Since our model lacks specific structural representation of basement membrane assembly dynamics, we could not simulate the FN generated “cleft forming force” which was hypothesized to be the primary cause for progressive clefts [10]. Therefore, we attempted to simulate the effect of this FN-actomyosin dependent “cleft forming” force through an energy function called focal point plasticity (FPP). This function establishes links between selected cells and regulates the distance between them, assigning an energy penalty for deviating from a target distance. As noted in Eq. 4, the penalty varies based on the target distance, and the λ term. To replicate the wedge-shaped cells in the cleft, we paired opposite cells on each side of the cleft, and set decreasing target distances for pairs deeper within the cleft. These target distances were determined by examining cleft depths from ex-vivo time-lapse images and measuring cleft width as a function of depth (Figure S1). We found that a target distance inversely proportional to the cleft depth approximated the observed shape. Modulating the λ term adjusts the strength of this cleft-opening/maintaining effect. Due to the fundamental role of actomyosin contractility in FN assembly, it can be viewed as modulating contractility levels within the cleft cells.

In case of cleft progression, the exact roles for actin contractility in force generation during progression is unknown and although phosphorylated NM-myosin II was detected in the OCCs [6], it is not known if OCCs contract by pulling on each other through the actomyosin bundles. So, we utilized FPPλ to assign lateral FPP links in the OCC layer between adjacent cells and additional vertical links and lateral links between cleft cells (Figures 3A, 3B). These lateral links in the OCCs helped control the shape of the boundary cells along with maintaining a constant epithelial boundary. We then utilized the lateral FPP links in the cleft cells to simulate the effect of this actomyosin dependent FN “wedge.” The varying target distances in the cleft region are manipulated dynamically to simulate the effects of a “clefting force” generated by continuous actomyosin-mediated FN assembly between the cleft cells as the cleft progresses inward.

### Designation of Cell Proliferation Properties

Previous work shows cell proliferation to be dispensable for cleft initiation [41], but to be required for cleft progression [6]. Although cytoskeletal contraction can induce cell proliferation [42], [43], in the CC3D environment, cell proliferation can be regulated separately from cell contractility. In the model we designated not only the percentages of mitotic cells but also their location within each epithelial cell subtype.

### Temporal Calibration of the GGH Model with Image Data

We ran initial simulations for an extended number of MCS steps to determine the range of MCS steps corresponding to the time frame encompassing cleft initiation through progression (Figure 1E). A termination value of 1500 MCS steps was selected, equating to a temporal conversion of 1 MCS≈48 seconds (Figure 3E, 3F). Figures 3A and 3B show the model at time 0 hrs (0 MCS) and time 20 hrs (1500 MCS), respectively.

### Establishing Initial Parameters for the Single Cleft Model

Within the CC3D environment, we established a set of base values for the five primary epithelial parameters included in this computational model under which cleft progression could occur (Table 1). To conduct a parametric search, we fixed the temperature (T) at 10. Due to its central role in the energy minimization step, modification of T impacts every other energy-based parameter. We vary T and select a fixed value that permits cells to fluctuate fluidly without becoming fragmented [27], consistent with previous observations that epithelial cells undergo dynamic movements during branching morphogenesis [10], [23]. This simulates a basal level of cell migration in both OCC and IPC epithelial cells. Interestingly, the random cell movement observed produces some exchange of cells between the OCC and IPC layer. With T at 10, we conducted a parametric search on these parameters: focal point plasticity (FFP λ), mitosis rate, mitosis location, cell-cell contact energy, and cell-matrix contact energies.

**Table 1:** CC3D parameters that were varied in the model and their biological significance in branching morphogenesis and cleft formation.

CC3D parameters to vary	Biological effect simulated	Experimental data - Effect seen in cleft formation	Unknown biological effects
Focal point plasticity λ (FPP λ)	Actin-myosin contractility in the cleft cells	Decreasing contractility prevents initiated clefts from progressing [6]	Increasing contractility.Increasing or decreasing contractility within cleft region
Mitosis rate (MR)	Epithelial cell proliferation – in outer columnar cells (OCC) and inner polymorphic cells (IPC)	Decreasing cell proliferation in the whole organ decreased cleft progression but not initiation [6]	Increasing mitosis rate.Varying mitosis rates in specific subsets of cells in OCC and IPC populations.
Cell-cell (CC) contact energy	E-cadherin-based cell-cell junctions	E-cadherin mRNA was found to be ∼6 fold lower in the cleft epithelium than in the bud epithelium [10] Global reduction of E-cadherin decreases branching morphogenesis [38]	Increasing E-cadherin protein activity.Increasing or decreasing E-cadherin levels in the cleft region
Cell-matrix (CM) contact energy	Cell-matrix adhesions	Decreased FN decreases cleft formation [5]. Lower FN assembly reduces cleft depth [22]	Increasing FN assembly levels in the cleft region

To yield a final cleft depth of 36 pixels in 1500 MCS (Video S3), we fixed these base values for the five parameters: Mitosis rate was set to 1% (per 100 MCS steps), evenly divided within OCCs and IPCs; FPP λ values in the OCCs and cleft cells was set at 10; cell-cell contact energy was set to 10 for cleft cells and 5 for all other cells; and cell-matrix adhesion in cleft cells was set to 3. Under these parameters, our model achieved an average cleft depth of 34.1 pixels, thereby yielding a spatial conversion of 1 µm = 1.06 pixels. Each simulation was run 100 times to ensure the consistency of the results given the stochastic nature of the GGH model. Figure 3F shows an example of the temporal evolution of cleft depths, achieving a 34.1 µm depth in 1500 MCS. With T value fixed at 10, we tracked 1725 individual cells in the base case simulation for 10 runs. The average net displacement was found to be 7.3 µm and the total path length was 94.6 µm (Figure S2). Thus the cell velocity was calculated to be 4.7 µm/hour and the meandering index to be 0.08 in the base model.

### Quantitative Analysis of Cleft Progression

**Figure 4:**
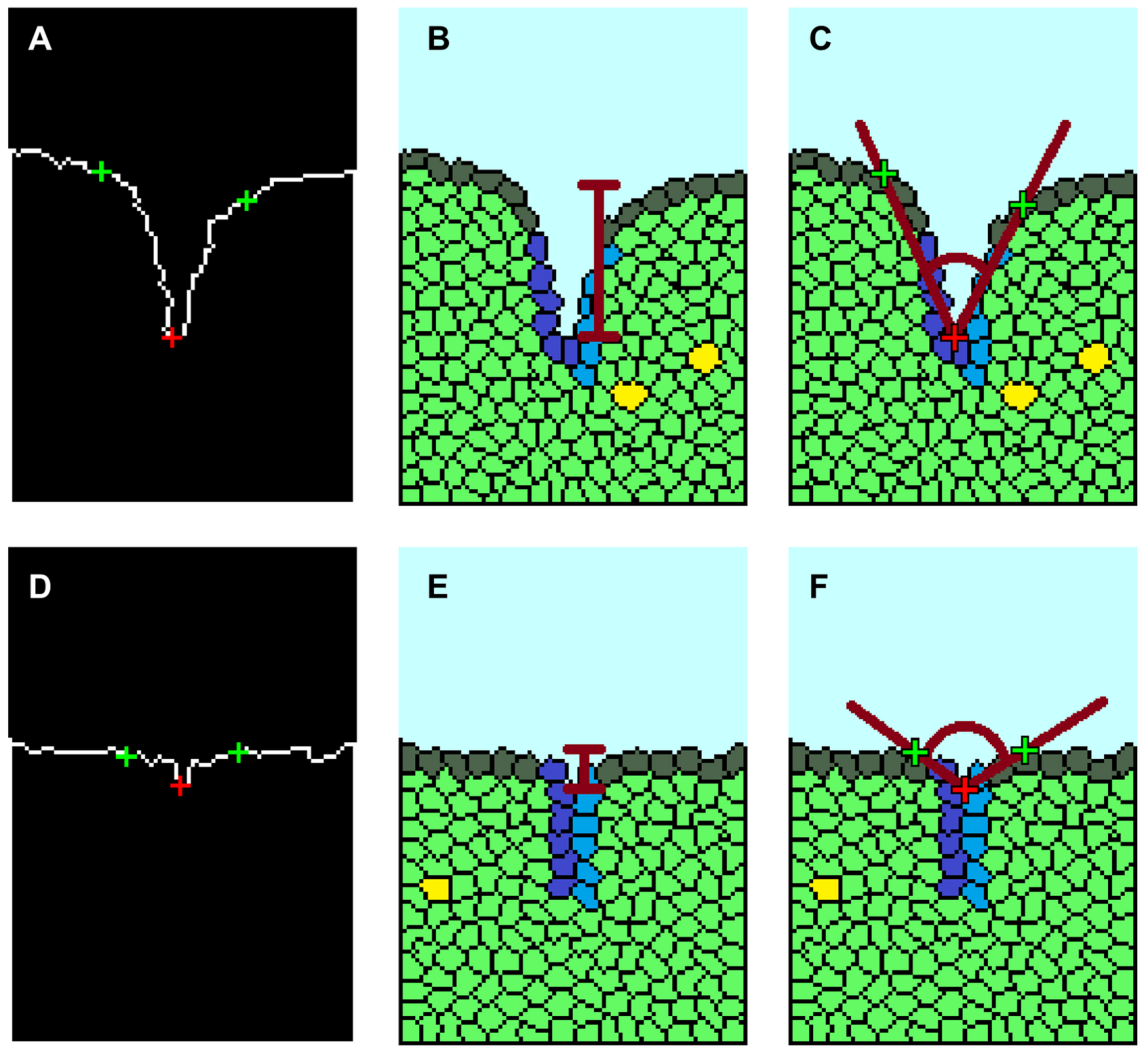
Quantitative analysis of cleft formation. A MatLab function was created for tracing the border of the local cleft. (a) The two cleft extremes were labeled in green and the cleft tip in red. A MatLab tracing of a successful cleft at 1500 MCS shows a (b) high cleft depth and (c) low spanning angle (red lines). (d) MatLab tracing of a non-progressive cleft at 1500 MCS shows (e) low cleft depth and (f) high spanning angle.

For quantitative and consistent methods to measure the quality of simulated clefts by comparison with equivalent measurements from organ explants, we developed descriptive cleft measurement indices – cleft depth, spanning angle, and tilt angle. First, the cleft center was located at the epithelial-mesenchymal boundary by examining the angle formed by each boundary point and its 8-distance neighbor on either side. As the deepest point of the cleft, the cleft center should have the lowest such angle value. The extrema are identified by using the mean-squared error (MSE) of the best-fit line for the boundary on each side of the cleft center; for each side, we progressively include points from the boundary until the MSE exceeds a predetermined threshold. The cleft center and extrema are shown in the example image in Figure 4A, 4D. Cleft depth is calculated as the distance from the cleft center to the midpoint of the line segment joining the two extrema (Figure 4B, 4E). Spanning angle is calculated as the angle formed by the line segments joining the cleft center to each extrema (Figure 4C, 4F). Clefts measuring less than 5 pixels in depth or exceeding 160° in spanning angle were discarded. The tilt angle is a measure of the perpendicularity of a cleft to the bud surface. It is calculated as the smaller of the complementary angles formed by the line segment between the extrema, and the line segment from the cleft center to the midpoint of the line segment joining the two extrema, as shown in Figure S3. Clefts with a tilt angle of less than 45° were labeled as “failed clefts”. The cleft categorization criteria were based on measured properties of clefts from multiple time-lapse images of organ explants.

### Building an Organ Level Model of Cleft Progression

**Figure 5:**
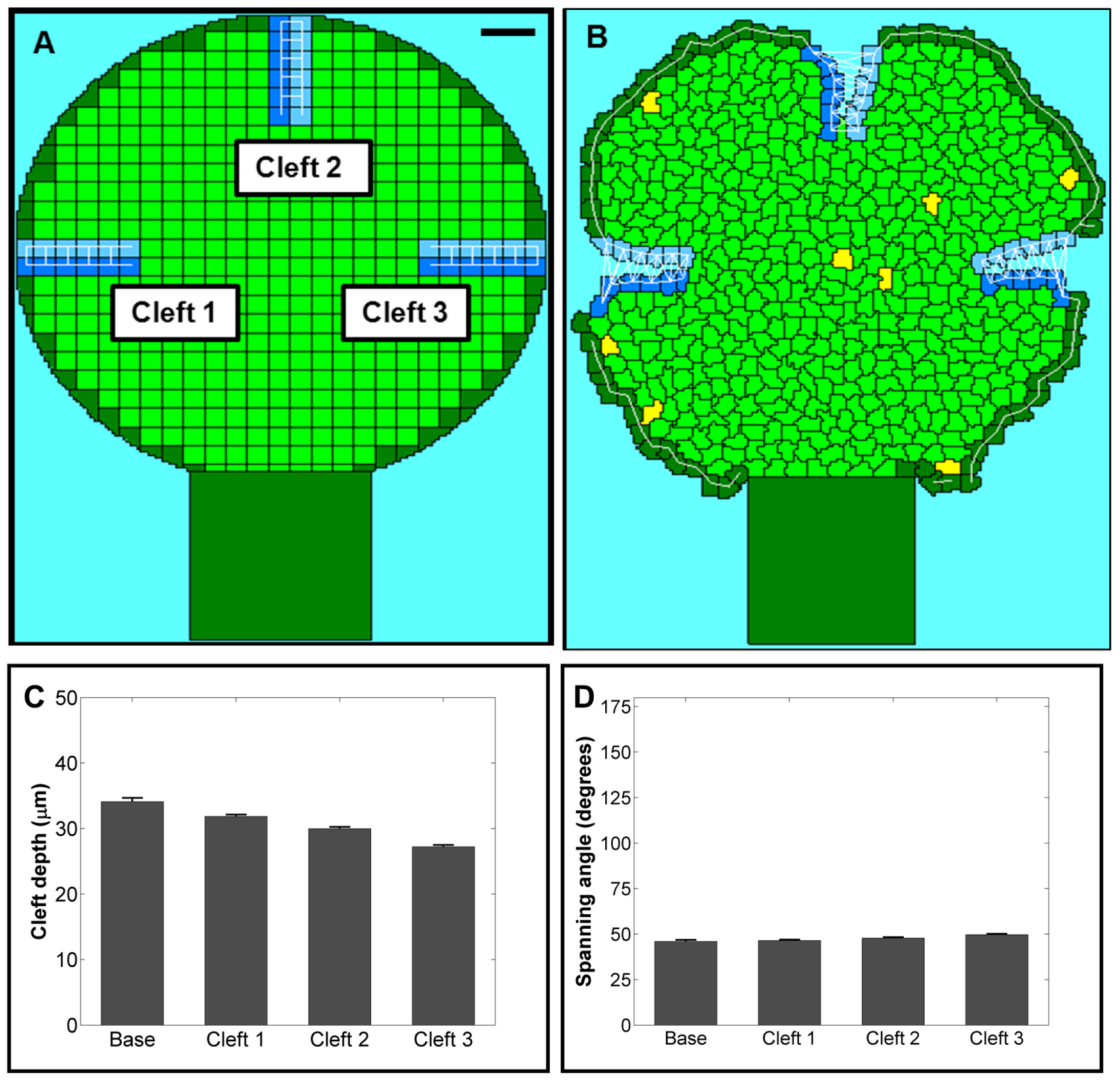
Organ level model. An organ-level simulation containing three ideally localized clefts is shown at (a) 0 MCS and (b) 1500 MCS (Scale = 50 µm). The results were quantified with (c) cleft depth and (d) spanning angle. A slight decrease in the average cleft depth and increase in the average spanning angle was observed relative to the single cleft model run under the same basal conditions.

In a developing salivary gland, multiple clefts form on the surface of the primary bud during branching morphogenesis, and they do not all form simultaneously. To determine if the progression of one cleft has an effect on adjacent clefts, we constructed a GGH-based salivary gland organ model consisting of a single bud with three equally-spaced clefts that progress simultaneously (Figure 5A) We ran 70 independent simulations with the same base case parameters that were used for the single cleft model, each for 1500 MCS (Figures 5A, 5B, Video S4). Quantitative analyses show that each individual cleft is comparable to those produced by the single local cleft model, albeit the average final cleft depth is slightly lower at (Figure 5C) 29.7 µm rather than 34.1 µm for the single cleft model. Correspondingly, marginally higher spanning angle values were observed compared to the base case. This result interestingly predicts that the behavior of clefts is somewhat dependent upon adjacent clefts. However, to focus on the cellular parameters necessary for progression of a single cleft, we used the single cleft model in all subsequent studies.

### Validation of the Single Cleft Model

**Figure 6:**
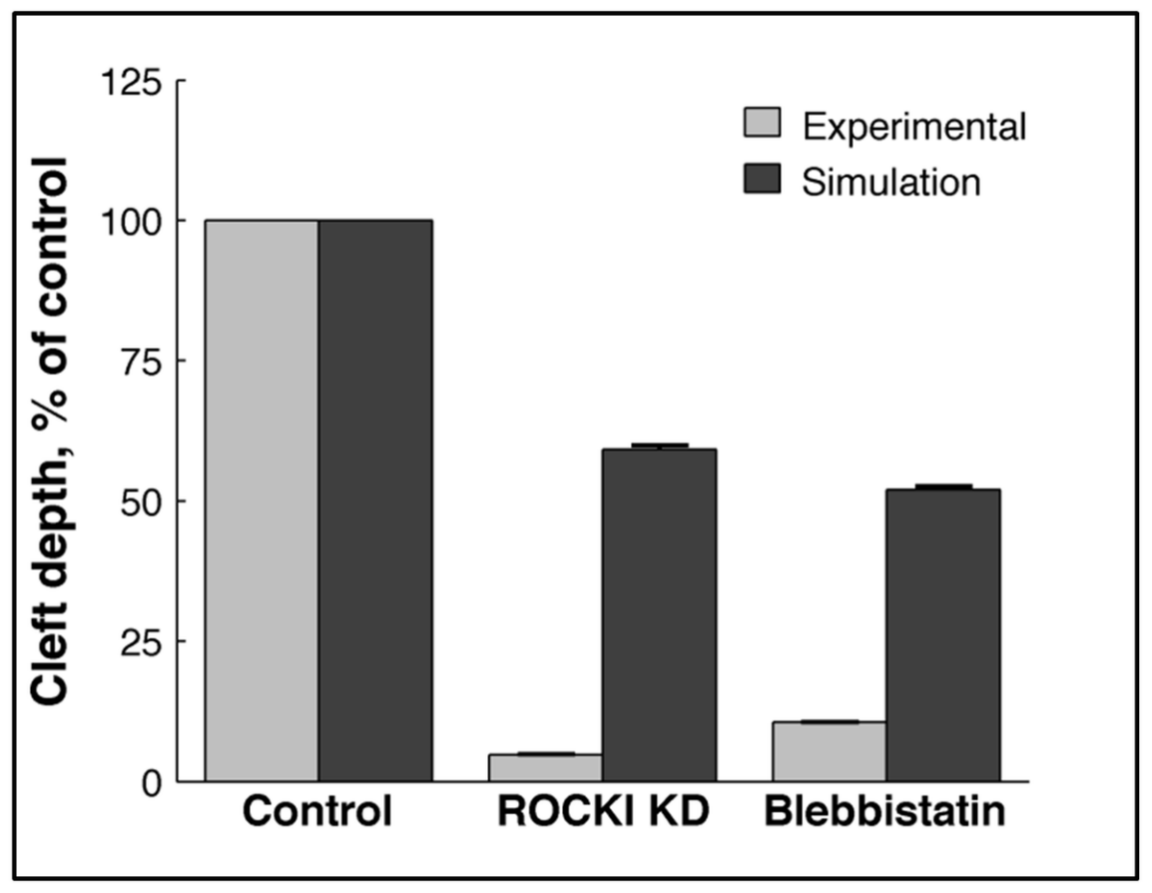
Validation of the single cleft model by comparison of predictions with experimental results for manipulation of ROCK. SMGs were cultured ex-vivo and treated with Y27632 (ROCK inhibitor) and blebbistatin (inhibitor of actomyosin contractility that lowers the affinity of myosin-actin interaction) for 24 hours. Cleft depths were measured using brightfield time-lapse confocal imaging and compared with cleft depths in GGH simulations of ROCK I knockdown (KD). ROCK I KD simulation consisted of a reduced FPP λ value (1) along with a reduced proliferation rate (0.05%). The simulation mimicking blebbistatin action was achieved using only a reduced FPP λvalue (1). Similar trends were observed in the simulations and the experimental results, indicating that the model effectively simulated the cellular effects of inhibition of signaling molecules.

Having built a cellular model replicating cleft progression, we ran simulations comprising combinatorial variations of two parameters to simulate a specific biological state. As cleft progression requires ROCKI signaling, which stimulates both actomyosin contractility and proliferation [6], we simulated these cellular conditions by reducing the lateral FPP λ values in the cleft cells from 10 to 1 to simulate reduced cellular contractility and correspondingly lowered the mitosis rates in all cells from 1% to 0.5%. We performed 100 simulations and quantified cleft quality using the three cleft measurement indices. As shown in Figure 6, the cleft depths were reduced by 40.8% in simulations and 94.7% in ex-vivo studies using 10 µM Y27632 treatments for 24 hours (Figure 6, Video S5). We also simulated the effects of blebbistatin, a pharmacological inhibitor that prevents high affinity interactions between actin and myosin to inhibit cleft progression but does not affect cell proliferation [6] using an FPP λ value of 1 in the cleft cells but without changing cell proliferation rate. We observed similar trends in the reduction of cleft depths: a 48% reduction with 20 hours of in silico simulation in comparison to 88.9% reduction in organ explants treated for 24 hours with 25 µM blebbistatin (Figure 6, Video S6). Interestingly, the computational model agrees qualitatively with the experimental data that cell contractility and mitosis affect cleft progression.

Use of the single cleft model to test hypotheses regarding the mechanisms of cleft progression-

### Hypothesis I: Cell Proliferation Drives Cleft Progression

**Figure 7:**
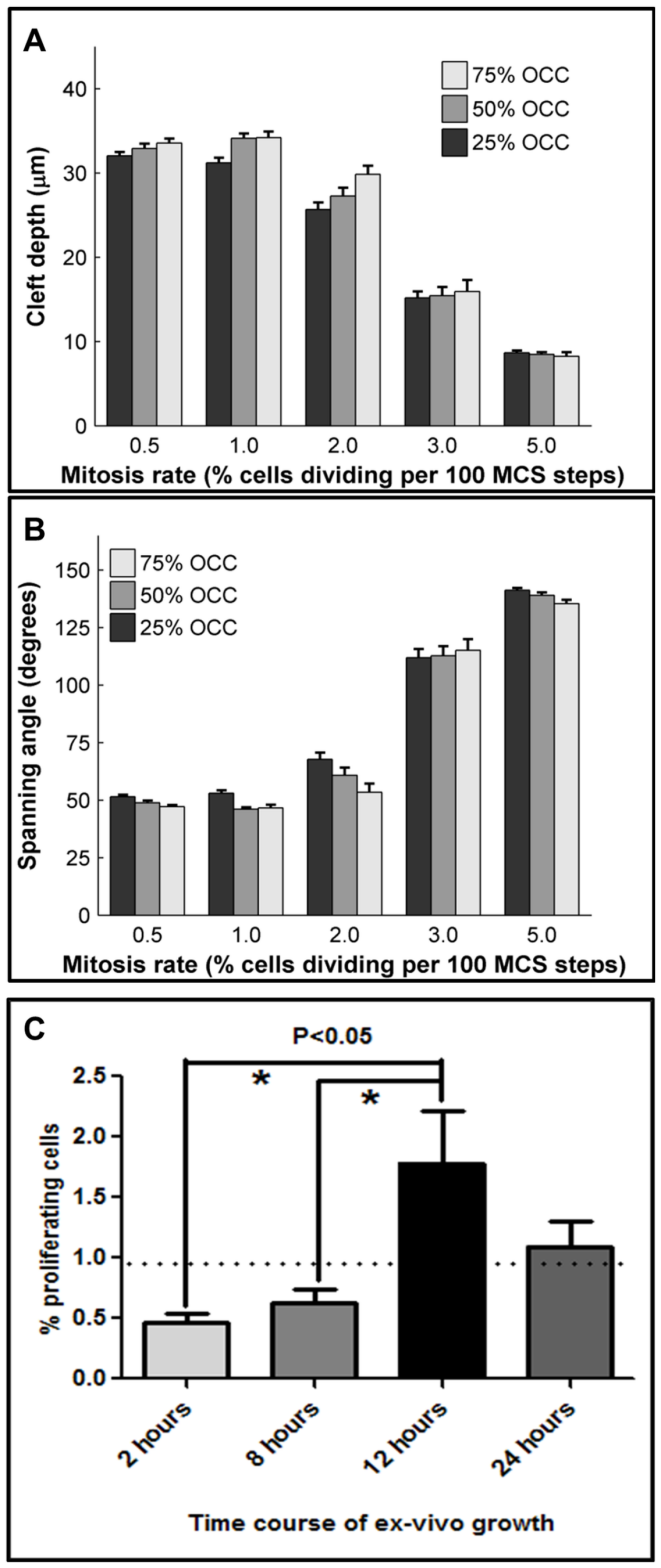
Effect of varying mitosis location on cleft progression. The location of proliferating epithelial cells was varied, with 25%, 50% or 75% mitosis occurring in the OCC population, while the MR rate was also varied from 0.5–5%. Results were quantified as (a) cleft depth and (b) spanning angle. The location of proliferating cells had an effect on cleft formation with 50–75% proliferation in the OCCs generally being more effective than 25%. (c) Image segmentation and analysis of confocal images acquired from pHH3 ICC and SYBR green-stained explants at four time points shows temporal changes in mitosis rate that produce an average epithelial mitosis rate of 0.99% between 2–24 hours of ex vivo culture. Dotted line denotes average mitosis rate (0.99%), ANOVA *P<0.05, n = 6.

Cell proliferation has long been understood to occur during branching morphogenesis. An early study indicated that cell proliferation was not required for salivary gland cleft formation [41], but later work demonstrated that although cleft initiation does not require cell proliferation, the biochemically independent step of cleft progression does require cell proliferation [6]. However, it has not been possible to experimentally increase the cell proliferation rate without affecting other cellular parameters. To test the sub-hypothesis that high cell proliferation rates are required for cleft progression, we performed simulations in which we tested increasing amounts of cell proliferation by varying the value for the GGH parameter, mitosis rate (MR). For this in silico experiment, we chose to assign the dividing cells equally in the OCC and IPC epithelial cells. We ran 100 simulations for 5 different values of MR, from 0.5% to 5%. Surprisingly, we found that high MR levels were inhibitory for cleft progression and that the best conditions for promotion of cleft progression were at a MR of 1%, where the cleft depth was the highest and the spanning angle was the lowest (Figures 7A, 7B). To experimentally validate the prediction of the single cleft model that 1% cell proliferation is ideal for cleft progression, we grew organ explants for 24 hours, and fixed a subset of tissues for immunocytochemistry with pHH3 to detect cells in M phase and staining with SYBR green to detect total nuclei at 2, 8, 12, and 24 hrs. Mitotic cells were detected in both the OCC and IPC layers, and the percentages of dividing cells were calculated from single confocal images for each tissue compartment (Figure 2E, 2F). Although the mitosis rate varied over the time period of the assay, the average mitosis rate was calculated to be 0.99% in the epithelial region (Figure 7C), as predicted by the single cleft model.

It has not been experimentally possible to manipulate cell proliferation rates in specific regions of the gland; therefore, it is not known if there is a regional preference for cell proliferation within the epithelium during cleft progression. Using the single cleft model, it is possible to test the sub-hypothesis that the OCCs proliferate preferentially over the IPCs. We performed simulations where we both varied the epithelial location of the proliferating cells and also varied MR. MR was set at 0.5%, 1%, 2%, 3%, or 5%, with the location for mitotic cells designated as 25%, 50%, or 75% in the OCC population (Figure 7). When 25% of the proliferating cells were located in the OCCs, we found that increasing the mitosis rate from 0.5% to 1% caused a minor decrease in cleft depth (32 µm to 31.2 µm) (Figure 7A). Further increases in MR to 2%, 3% and 5% decreased cleft depths to 25.7 µm, 15.2 µm, and 8.7 µm, respectively. Interestingly, when 50% or 75% of the mitotic cells were located in the OCC region, by increasing MR from 0.5% to 1%, a slight increase in cleft depth from 32.3 µm to 34.1 µm was observed; but further increases in rates to 2%, 3%, and 5% caused progressive decrease in depths, irrespective of the location of the dividing cells. The trends in spanning angle are shown in Figure 7B. Thus, 1% MR with 50–75% of the cells located in the OCC region was found to be the optimal condition for cleft progression.

### Hypothesis II: Cellular Contractility Drives Cleft Progression

In previous work, we demonstrated that ROCKI is required for cleft progression through modulation of actomyosin contractility [6]. ROCKI was required for phosphorylation of NM myosin II to stimulate contractility and down-regulation of cellular contractility with blebbistatin similarly reduced cleft progression. Pharmacological inhibitors cause a global reduction of actomyosin contractility, making it impossible to assess the effect of cellular contractility specifically in the cleft cells. Actin-based contractility is responsible for dynamic cell movements and FN assembly by the cleft cells, and so we modulated the strength of the lateral FPPλ links in the cleft cells to test the hypothesis that actomyosin contractility in the cleft cells is required for cleft progression. In the cleft region, we assigned vertical FPP links between cleft cells and also lateral links between the adjacent cleft cells on either side of the cleft. Also, each pair of cleft cells was assigned a different target distance, with a lower target distance being set for cells deep within the cleft. During the course of simulation, each pair of cleft cells strives to acquire the set target distance, and after 1500 MCS, under unaltered cell and matrix contact energy settings, FPP energy is solely dictated by λ values since the distance deviation for each pair of cleft cells remain almost constant for varying λ values. Thus, by altering the lateral FPPλ values in the cleft cells, we aimed to vary actomyosin-based cellular contractility specifically in these cells to assess its effect on cleft progression.

**Figure 8:**
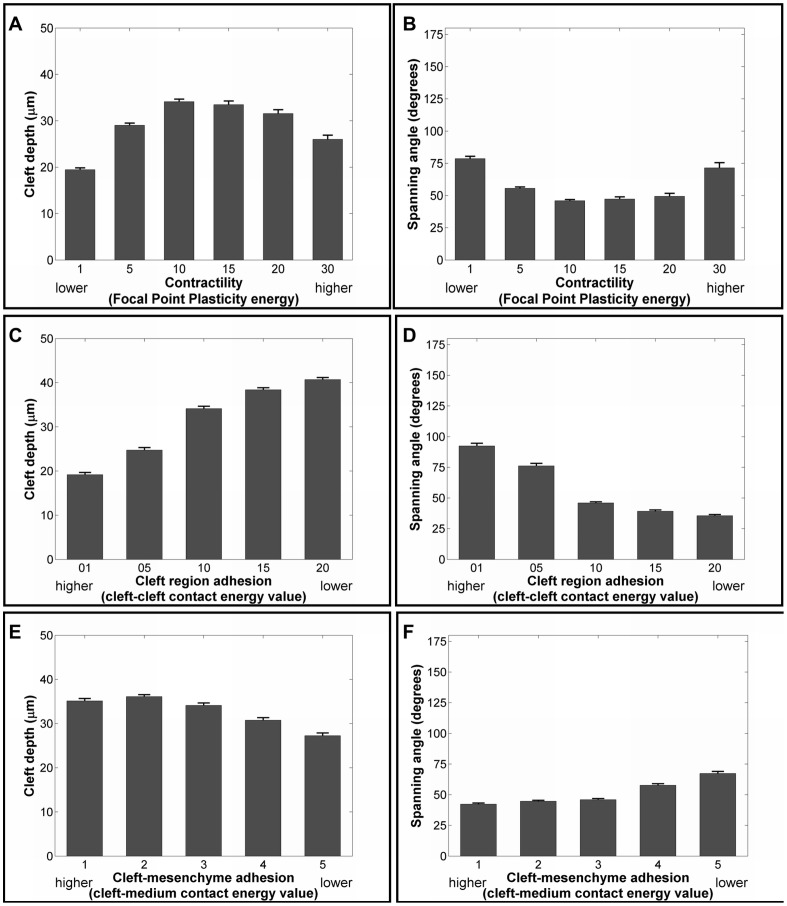
Effect of varying cell contractility, cell-cell adhesion, and cell-matrix adhesion strength on cleft progression. FPP λ values were varied between 1–30, and effects on cleft progression were quantified with (a) cleft depth and (b) spanning angle. FPP λ values lower or higher than 10 are detrimental to cleft progression. Cell-cell (CC) adhesion strength in the cleft region was manipulated by increasing the cell-cell contact energy to mimic E-cadherin-based adhesions and evaluated with (c) cleft depth and (d) spanning angle. Decreasing cell-cell adhesion in the cleft region (increased contact energy) resulted in deeper clefts with lower spanning angles. Modulation of cell-ECM (CM) junctional strength by increasing cell-matrix contact energy was monitored by (e) cleft depth and (f) spanning angle. Increasing cell-matrix junctional strength affects clefts quality marginally with greater cleft depth and lower spanning angle.

To test the hypothesis that contractility in cleft cells is required for cleft progression, we varied the lateral FPP λ values that hold the cleft cells together, between 1 and 30. A cleft depth of 34.1 µm and a spanning angle of 46.0° was found for FPP λ = 10, whereas lowering FPP λ values to 5 or 1 caused the average cleft depth to decrease to 29.0 and 19.5 µm respectively (Figure 8A) with associated increases in spanning angle (Figure 8B). This manipulation mimicked the effect of decreasing actomyosin contractility, as performed experimentally using blebbistatin. Interestingly, when we used higher values of FPP λ such as 15, 20, and 30, progressively lower cleft depths (33.5, 31.6, and 26.0 µm) and higher spanning angles (47.3, 49.3, and 71.5°) were observed, which has not been experimentally tested. When no FPP links were used, shallow clefts were formed with an average cleft depth of 12.6+/−3.94 µm and spanning angle of 99.9° (Video S2). The single cleft model thus predicts that actomyosin contractility in the cleft is essential for cleft progression and that a moderate level of this cellular contractility favors cleft progression, with low contractility being more detrimental to cleft progression than high contractility.

### Hypothesis III: Lowered Cleft-Cell Adhesion and Increased Cleft Cell-Matrix Adhesion Drives Cleft Progression

It was previously reported that loss of E-cadherin-containing cleft-cell adhesions and gain of fibronectin-driven cell-matrix adhesions within the cleft region are required for cleft progression, and further that an epithelial-mesenchymal transition occurs in a subset of cells at the base of the cleft to facilitate cleft progression [5], [22]. Although experimental manipulations have been performed to examine the effect of decreasing E-cadherin-based cleft-cell adhesions [10], [38] and to increase or decrease cell-matrix adhesions [5], [6] these manipulations have been performed with whole organ explants or with whole epithelial rudiments grown in an artificial matrix and the requirement for these changes specifically within the cleft region has not been possible to address. With the single cleft model, it is possible to manipulate cleft-cell and cell-matrix adhesion strengths within a subset of cells in the cleft region by running simulations at multiple values.

To recapitulate a progressive EMT occurring in the progressing cleft, we assigned a low cell-matrix adhesion value of 3 and a higher cell-cell adhesion value of 10 in the cleft cells forming the cleft wall, whereas all other cell-cell contact energies remained at 5. These values are assigned at the onset of simulation, and in each pair of cleft cells, cell-cell adhesions are replaced by epithelial-matrix adhesions during the temporal progress of cleft deepening. In order to preserve the epithelial organization of the OCC and IPC relative to the matrix, we assign a lower energy penalty between OCCs and a higher penalty for IPC-matrix. When cleft-cell adhesion was decreased (raised contact energy), cleft depths increased beyond 34.1 µm to 38.4 (CC value = 15) and 40.7 µm (CC value = 20) (Figure 8C) with corresponding spanning angle measurements that decreased from a base value of 46.0° to 39.3° and 35.6° (Figure 8D), respectively, following 100 simulations. Interestingly, increasing cleft-cell adhesions (lowering contact energy values to 5 and 1) caused shallower clefts with depths of 24.7 µm and 19.2 µm and increased spanning angles to 76.2° (C-C value = 5) and 92.4° (C-C value = 1). Thus, the single cleft model predicts that low cell-cell adhesion strengths within the cleft are most beneficial for cleft progression.

It has also not been possible to determine experimentally whether it is the loss in cleft-cell adhesions or the increase in cell-matrix adhesions that occurs in progressive clefts that has the most significant effect on cleft progression. In the single cleft model, we varied the cleft-matrix contact energy from 1 to 5 and ran 100 simulations with each value. Unexpectedly, variations in the cell-matrix contact energy values had minimal effects on cleft progression. Higher cleft cell-matrix contact energy values (lowered adhesion) yielded slightly shallower clefts (30.8 µm for CM = 4 and 27.3 µm for CM = 5) than the base value of 3 (Figure 8E), with corresponding changes in spanning angles (Figure 8F). Lower cleft cell-matrix contact energies (increased adhesion) yielded marginally deeper clefts (36.1 µm for value = 2 and 35.1 µm for CM = 1), with corresponding changes in spanning angles, than obtained with the base value of 3. Thus, the single cleft model predicts that for efficient cleft progression in the cleft region, a low cell-cell adhesion value is required more so than specific levels of cell-matrix adhesion; however, higher cell-matrix adhesion levels are somewhat beneficial for cleft progression.

### Relative Contribution of Cellular Parameters for Cleft Progression in Branching Morphogenesis

**Figure 9:**
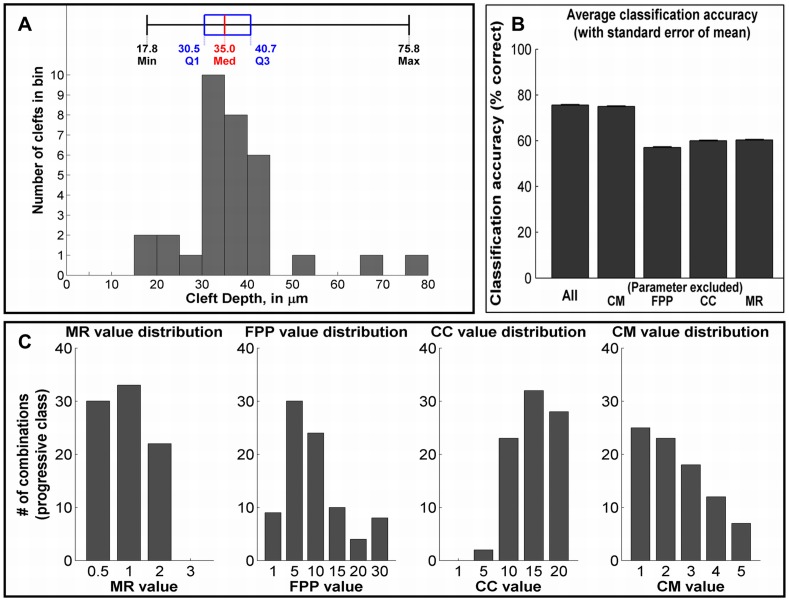
Classification analysis. (a) Distribution of cleft measurements from SMG organ explant image data. Classes for parameter combinations were assigned as follows: failed, <17.8 µm; non-progressive, 17.8–30.5 µm; progressive, 30.5–40.7 µm; super-progressive, >40.7 µm. (b) Cross-validated classification accuracy on test set for the full parameter combination (CC, CM, FPP and MR), and exclusion of a single parameter CC, CM, FPP, or MR. A larger decrease in accuracy indicates greater importance. (c) Parameter value distributions within the progressive class. Peaks indicate higher importance for that value in progressive cleft formation.

Although multiple studies have been performed to assess the importance of individual cellular factors in the process of branching morphogenesis, it is not possible to rank the importance of these cellular factors using experimental methods alone. Using the single cleft simulation model, we varied each of the four parameters independently for a total of 600 parameter combinations. Each parameter combination was simulated 40 times, and classified into one of four categories based on the majority result. These cleft classes were designated based on the distribution of measurements derived from time-lapse data, and labeled “failed,” “non-progressive,” “progressive,” and “super-progressive.” Failed (F) clefts did not stabilize and regressed back to the epithelial boundary and non-progressive (NP) clefts stabilized but failed to progress. Progressive (P) clefts fall within the normal size range of clefts measured from time-lapse data, whereas super-progressive (SP) clefts exceed the average size. The depths that each class corresponds to are shown in Figure 9A. Out of the 600 parameter combinations, we obtained 275 F, 188 NP, 85 P, and 52 SP combinations in each of the cleft classes.

The number of stabilized versus progressive clefts, for each parameter variation have been outlined in Figure S5 and in Tables S1 and S2. The proportion of F, NP, and P clefts defined the limits of our parametric search for each hypothesis. For instance, the range of FPP λ values was chosen after assessing the number of progressive clefts (P) obtained from each variation. Figure S6B shows that at λ value 0.5, no P was obtained. Hence the lower limit for FPP λ variation was set to 1. The number of P decreased with increasing contractility and a higher value of 30 was set as the upper limit of the range. Also from Table S1, the number of F and NP increased highly at λ value 30, and beyond 30 there were almost no P, with mostly destabilized failed clefts.

To assess the relative importance of each GGH parameter, we measured how accurately a classifier could predict the cleft class of a parameter configuration, with the expectation that parameters that have a high importance in cleft progression should also serve as good predictors of cleft class (simulation outcome). Conversely, when removed as a feature, the absence of such a parameter should have a strong negative impact on the classification accuracy. Using a radial basis kernel SVM classifier, we were able to achieve 75.6% accuracy when the classifier was provided with all four GGH parameters as features. We then attempted classification using the remaining 14 possible combinations of three, two, or one parameter. The cross-validated training and testing accuracies and decrease in accuracy relative to the full parameter set are reported in Table 2. The testing accuracy for combinations where a single parameter was removed is also shown in Figure 9B. Individual removal of the three parameters MR, FPP λ, and CC resulted in similar drops in classification accuracy of 15.3%, 18.5%, and 15.6%, respectively. The removal of cellular contractility (FPP λ) had a marginally higher impact on the classification accuracy than cell-cell adhesion (CC) and mitosis rate (MR). In contrast, omitting CM resulted in a classification accuracy of 75.0%, which is only a 0.6% decrease from the 75.6% accuracy level obtained with all four features, suggesting that the contribution of cell-matrix adhesions are the least significant contributor to cleft progression in our model. Thus, our analysis that considers the drop in classification accuracy as the metric for importance suggests that FPP λ is the most significant contributor to cleft progression in our model, followed closely by CC and MR.

**Table 2:** Classification accuracy table.

Classification Parameters	Training accuracy (%)	Testing accuracy (%)	Percent decrease in testing accuracy from Case I *
Case I	88.3±1.7	75.6±2.9	0.0
CC, FPP, MR	83.3±1.9	75.0±3.0	0.6
CC, CM, MR	64.9±2.1	57.1±3.6	18.5
CM, FPP, MR	67.2±2.1	60.0±3.5	15.6
CC, CM, FPP	71.2±2.1	60.3±3.4	15.3
CC, CM	64.6±2.1	60.7±3.4	14.9
CM, FPP	61.0±2.1	57.0±3.4	18.6
CC, FPP	47.8±1.8	37.5±3.8	38.1
CM, MR	65.4±2.2	59.0±3.5	16.6
CC, MR	49.5±1.9	42.5±3.2	33.1
FPP, MR	50.0±2.0	41.4±4.0	34.2
MR	42.9±1.6	37.9±3.5	37.7
FPP	47.1±2.0	44.3±3.4	31.3
CC	45.8±1.9	42.8±3.6	32.8
CM	33.6±1.6	27.2±3.8	48.4

All comparisons are made with respect to the Case I when all parameters are included.

* Case I = CC, CM, FPP and MR are included, where CC = Cell-Cell contact energy, CM = Cell-Matrix contact energy, FPP = FPP λ value, MR = Mitosis Rate.

After classifying each parameter set into the four cleft types, we questioned which specific parameter values are important for achieving clefts that fall into the data-driven normal cleft range, described as “progressive” clefts (30.5–40.7 µm ∼85 out of the 600 simulations). The distributions of FPP, CC, MR and CM values of all clefts falling into the progressive class are shown in Figure 9C. The results indicate that the conditions to form “progressive” clefts vary slightly from the conditions required to form clefts, in general: (i) the optimal FPP λ value was 5, rather than 10 (base case of 34.1 µm cleft depth), indicating that a slightly lower value of cleft cell contractility is sufficient for progressive clefts. (ii) CC values peaked at 15 and 20, showing that a lower level of epithelial adhesion favors cleft progression in the “progressive class” than in the base case category where the deepest clefts peaked at a value of 10. (iii) CM adhesion values did not peak particularly at any value, but a lower value (∼1) promoted cleft progression, whereas a value of 3 was optimal for the base case. The optimal MR was similar to that predicted by the base case cleft categorization. This analysis also indicated that CM had the lowest impact on cleft progression.

## Discussion

We describe here the first cell-based model of salivary gland branching morphogenesis, which is able to recapitulate many crucial epithelial cell behaviors and make predictions regarding the manipulation of these behaviors on the outcome of the tissue structure, thus spanning two biological scales. Since organ formation is a complex process that encompasses several conserved molecular, cellular, and genetic mechanisms that cooperatively aid in the formation of tissue structure, it is difficult with experimental manipulations alone to identify critical factors contributing to the overall morphogenetic process. Using the single cleft GGH-based model, we were able to assess the relative quantitative importance of various cellular parameters in the process of cleft progression and found that cleft cell contractility was comparatively the most significant cellular contributor to cleft progression, followed closely by cell-cell adhesion and mitosis rate, with cell-matrix adhesions showing less significant contributions to cleft progression. It is particularly significant that actomyosin contraction, the biological effects of which are closely mimicked by the GGH term, focal point plasticity (FPP λ), was the most crucial contributor to cleft progression in this model, thus supporting our prior experimental results indicating that actomyosin contractility is essential for cleft progression [6].

We mimicked the effects of actin contractility by establishing FPP links in the cleft cells both laterally and vertically. Cortical actin microfilaments run along the cell perimeter in SMG epithelial cells, and together with myosin provide tensile forces in and between the cells [42], [43]. In the GGH model, FPP links establish a similar kind of restraint to inter-cell dynamics. The λ value dictates the strength of these connections. Thus, upon varying FPP λ in the cleft cells, there was a biphasic response of cleft depth to strength of contractility. Previously, cleft progression was studied experimentally only with reduced actomyosin contractility, which resulted in non-progressive clefts [6]. The effect of increased contractility on submandibular gland branching morphogenesis has not yet been studied, but in lung morphogenesis, a general Rho activator caused a biphasic response to branching [44]. Although the highest dose of the Rho activator increased cellular contractility, it decreased the number of buds, in support of the idea that the effect of contractility on cleft progression is biphasic.

Modulation of the distribution of proliferating cells is not easily addressed experimentally. Upon varying proliferation rates and locations, our *in silico* results indicate that a low rate of cell division is conducive for cleft progression and the dividing epithelial cells should either be equally divided between the OCCs and the IPCs or located primarily in the outer cells. Increasing cell proliferation levels, irrespective of the location of the dividing cells, caused a decrease in cleft depth. These results apparently conflict with experimental studies demonstrating that growth factors such as FGF, EGF, and PDGF [8], [9], [16]–[19], [40], [45]–[47] that promote branching morphogenesis of organ explants by increasing proliferation through various complex regulatory networks. However, most of these studies did not specifically examine cleft progression per se, but found that proliferation is generally important for increased bud formation, ductal outgrowth, and regulating expansion/maintenance of progenitor cell populations. Our model does not address ductal outgrowth or specific progenitor cell populations, so modeling of these more complex events requires the addition of more complexity to our model [48], [49].

We developed the salivary gland cleft model to be as realistic as possible given the current limitations of the GGH modeling environment. The model was built with 6×6 pixel cells that were structured on a regular lattice, organized as two epithelial cell layers - the outer and the inner cells along with pre-ordained cleft cells in the outer layer. An oversimplification of the model is that the cell shapes are not accurately represented, as the shapes of epithelial cells are known to be irregular during early development [10], [22]. Since cell placement and cell shape changes are essential components of multiple developmental processes [33], [34], [50], it will be informative in future studies to utilize cell-graphs as a quantitative tool to define the accurate placement of cells into a GGH model, thus allowing us to accurately model the cell shape changes in correlation with experimental cellular events. The previously reported dynamic, irregular shape of cells in developing salivary glands likely relates to their movement during early development [10]. This movement may be facilitated by weak E-cadherin-mediated cell adhesions. In our local cleft model, fixing temperature (T) at 10 provided us with lower velocity, displacement and meandering index values than those previously calculated [10]. Increasing T in the GGH model allows cells to change their shape more freely; however, it does not simulate the extensive cell movements previously observed in embryonic glands using time-lapse imaging [10]. Future improvements to the model will include more accurate representation of cell shapes and more accurate modeling of the kinetics of cell motility in the epithelium.

Although loss of cleft cell-cell adhesion is closely associated with progression of cell-matrix adhesions, our feature selection results indicate that cell-matrix adhesions are not important for cleft progression, which may result from inadequate modeling of the basement membrane properties. Several biological studies have demonstrated a role for basement membrane proteins including fibronectin [5], [6], [10], collagens [45], [47], laminin α5 [9], and perlecan [19] in branching morphogenesis. Previous studies have observed collagen III to be accumulated in the narrow cleft base region [47]; thus, a model was proposed where interstitial collagen secreted by mesenchymal cells was proposed to initiate clefts that were stabilized through GAGs, resulting in accelerated proliferation. Structural representation of basement membrane components in future models will make it possible to model cleft initiation, which was not addressed in this study. Further research to combine the epithelial cellular factors along with assembly of secreted matrix proteins, needs to be conducted to so that cleft initiation, stabilization, and progression can be studied in a synchronized fashion.

The mesenchyme is complex and also contains both developing nerves and blood vessels along with fibroblastic mesenchymal and progenitor cells within an elaborate extracellular matrix. Components of the mesenchyme are important for morphogenesis as mesenchymal-epithelial interactions are required [2], [3], [32], [51]. Our recent study using cell-graphs uncovered a previously undetected rearrangement of mesenchymal cells in ROCK inhibitor-treated glands relative to untreated controls, suggesting that mesenchymal rearrangements impact branching morphogenesis [33]. Recent work has investigated the dynamics of epithelial cell progenitor populations in developing salivary glands [32], [49]. Since the GGH model is capable of modeling reciprocal interactions between multiple cellular subtypes in pattern formation and disease progression [52], [53], [54], it will be informative in future work to use GGH modeling to evaluate contributions of progenitor cell populations and epithelial cell subtypes to morphogenesis. Thus, the modeling of specific epithelial and mesenchymal cell subtypes into future modeling work will make it possible to more accurately assess the cellular mechanisms driving branching morphogenesis.

Thus, this study provides a realistic model of one of the significant events in salivary gland organ development – cleft progression. Various cellular factors that affect this morphodynamic pattern formation have been explored in detail, and biological validation has been provided wherever possible. Although manipulations of genes and protein functions using organ explants has provided insight into the molecular mechanisms driving branching morphogenesis, there are many experimental manipulations that cannot be performed with either ex-vivo explants or in-vivo organisms due to technical impossibilities or to limited resources. In silico analysis of multifactorial developmental events circumvent this disadvantage and provide us with crucial molecular clues that can be investigated using experimental biology, thus improving our understanding of the complex process of organogenesis.

## Supporting Information

Figure S1 **Width to depth ratio calculated in a progressing cleft.** (a) The cleft area was selected and segmented (b) to calculate the cleft depth (D). (c) the width (W) of the cleft was found to be inversely related to the depth of the cleft through the Equation: W = [402/(D+11)]−5.
(TIF)

Figure S2 **The migratory properties of epithelial cells in the GGH single cleft model.** (a) 1725 epithelial cells were tracked, and a majority of the cells were found to have 6–8 pixels displacement. The mean displacement was 7.3 µm. (b) Majority of cells travelled a total path length of 100–110 pixels with the average length traversed as 94.6 µm.
(TIF)

Figure S3 **Tilt calculation.** The tilt angle was measured as the smaller of the complementary angles formed by the line segment between the extrema, and the line segment from the cleft center to the midpoint. This is used to measure the relative alignment of the clefts to the bud surface. Clefts with a tilt angle of less than 45° were eliminated as “failed clefts”.
(TIF)

Figure S4
**Determination of relationship between focal point plasticity λ value, target distance D, and cleft-ECM contact energy (CM).** (a) A simplified simulation was initialized with two 6×6 cells subjected to area and perimeter constraints, (b) A simulation was run for 1000MCS for varying values of D, CM and λ, and final cell distances were recorded. (c) Final stage of cell separation. (d) For each value of D selected, the λ and CM values required to achieve separation were saved and plotted. For these simulations, cell-cell contact energy value (CC) was kept constant at 10. A surface was fitted to these points in the form: 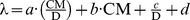 This equation approximates the λ value required to achieve separation between two linked and opposing cleft cells under conditions in the single cleft simulation. It was used to select a range of focal point plasticity λ values that allowed us to examine the interplay between cleft-cell adhesion, cell-matrix adhesion, and mitosis rate.(TIF)

Figure S5 **Ratio of progressive to non-progressive clefts obtained during parametric search.** (a) Mitotic rate (MR) variation from 0.5% to 5% (b) Focal point plasticity λ (FPP) variations from 0.5 to 30 (c) Cell-cell (CC) contact energy variation from 1 to 20 and (d) Cell-matrix (CM) contact energy variations from 1 to 5. For all the parameters, the corresponding ranges have been chosen based on the number of progressive clefts obtained in comparison to the number of non-progressive clefts and failed clefts.
(TIF)

Table S1 **Cleft categorization during parametric search enabling choice of ranges of values for mitosis rate and focal point plasticity λ.** Clefts were categorized as failed, progressive and non-progressive based on cleft depths measured from time-lapse videos of ex-vivo cultured explants.
(DOCX)

Table S2 **Cleft categorization during parametric search enabling choice of ranges of values for cell-cell and cell matrix contact energies.** Clefts were categorized as failed, progressive and non-progressive based on cleft depths measured from time-lapse videos of ex-vivo cultured explants.
(DOCX)

Video S1 **20 hour time-lapse confocal movie of an E12 epithelial rudiment used for cleft depth measurements.**
(AVI)

Video S2 **Example of a local cleft simulation with FPP = 0 [T = 10, Cell-cell adhesion at cleft cells = 10, Cell-matrix adhesion at cleft cells = 3, Mitosis rate = 1%, Mitosis location = 50% OCC, /50% IPC].**
(AVI)

Video S3 **Example of a GGH local salivary gland cleft simulation using base parameters: [T = 10, Cell-cell adhesion at cleft cells = 10, Cell-matrix adhesion at cleft cells = 3, Mitosis rate = 1%, Mitosis location = 50% OCC/50% IPC, FPP λ (cleft cells) = 10].**
(AVI)

Video S4 **Example of salivary gland organ level GGH simulation using base parameters [T = 10, Cell-cell adhesion at cleft cells = 10, Cell-matrix adhesion at cleft cells = 3, Mitosis rate = 1%, Mitosis location = 50% OCC/50% IPC, FPP λ (cleft cells) = 10].**
(AVI)

Video S5 **Example of a local cleft simulation under conditions simulating ROCK I inhibition [T = 10, Cell-cell adhesion at cleft cells = 10, Cell-matrix adhesion at cleft cells = 3, Mitosis rate = 0.5%, Mitosis location = 50% OCC/50% IPC, FPP λ (cleft cells) = 1].**
(AVI)

Video S6 **Example of a local cleft simulation under conditions simulating blebbistatin treatment [T = 10, Cell-cell adhesion at cleft cells = 10, Cell-matrix adhesion at cleft cells = 3, Mitosis rate = 1%, Mitosis location = 50% OCC/50% IPC, FPP λ (cleft cells) = 1].**
(AVI)
