# Supplementary figures and images for: Cell-Based Multi-Parametric Model of Cleft Progression during Submandibular Salivary Gland Branching Morphogenesis
Source: PLoS Comput Biol. 2013 Nov 21;9(11):e1003319. doi: 10.1371/journal.pcbi.1003319 (PMC3836695; doi:10.1371/journal.pcbi.1003319)

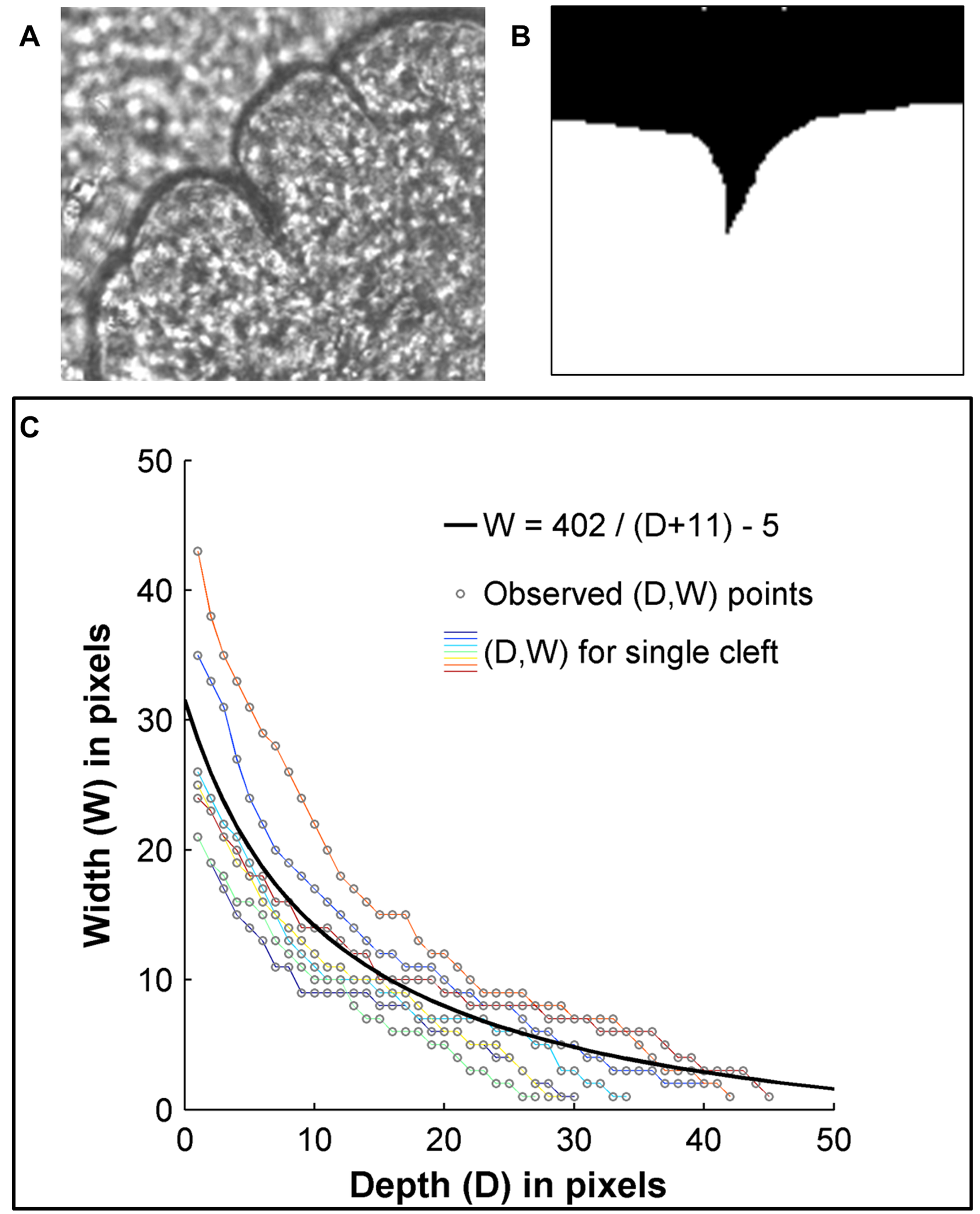

Supplement: Figure S1 — Width to depth ratio calculated in a progressing cleft. (a) The cleft area was selected and segmented (b) to calculate the cleft depth (D). (c) the width (W) of the cleft was found to be inversely related to the depth of the cleft through the Equation: W = [402/(D+11)]−5. (TIF) [file pcbi.1003319.s001.tif]

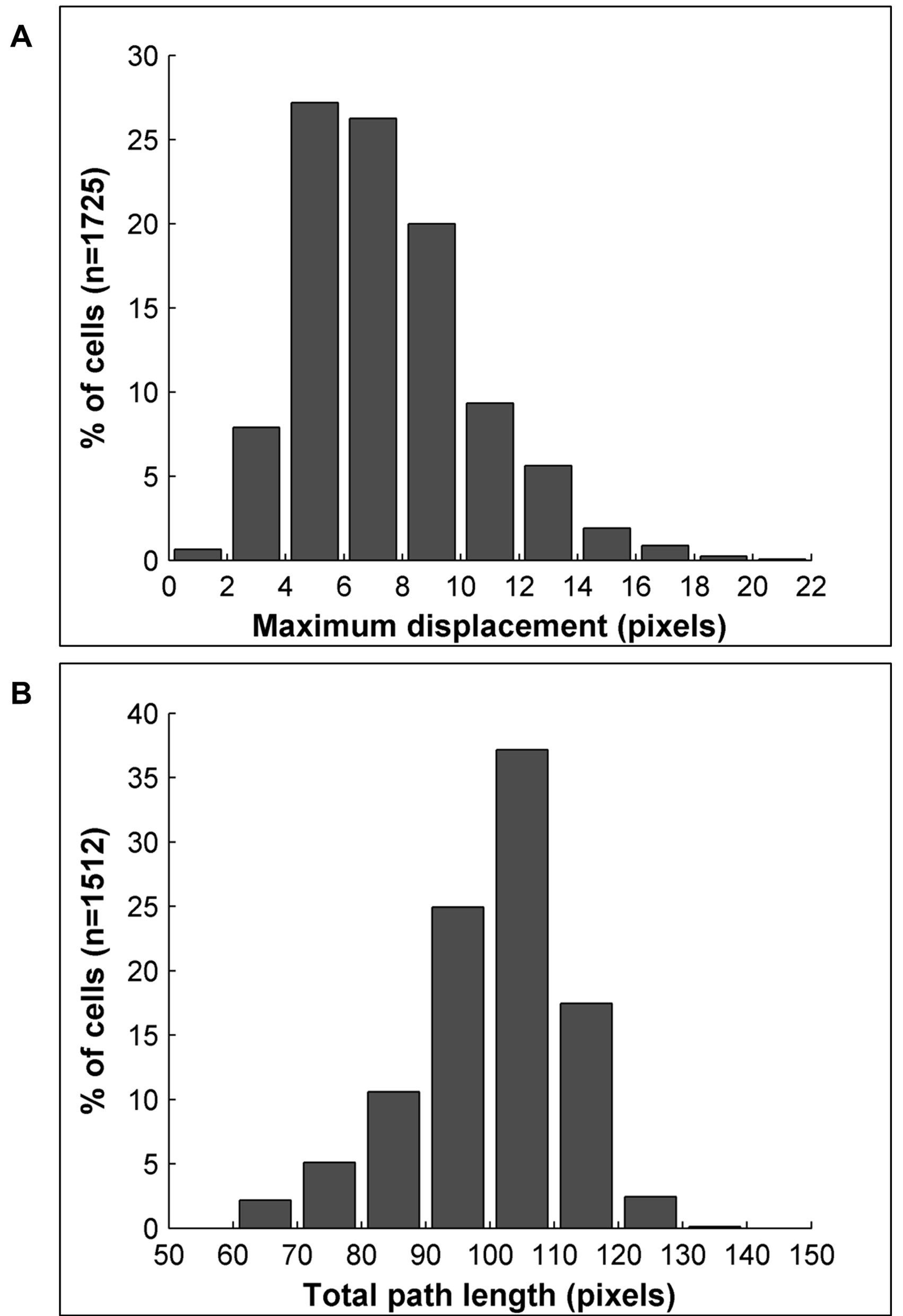

Supplement: Figure S2 — The migratory properties of epithelial cells in the GGH single cleft model. (a) 1725 epithelial cells were tracked, and a majority of the cells were found to have 6–8 pixels displacement. The mean displacement was 7.3 µm. (b) Majority of cells travelled a total path length of 100–110 pixels with the average length traversed as 94.6 µm. (TIF) [file pcbi.1003319.s002.tif]

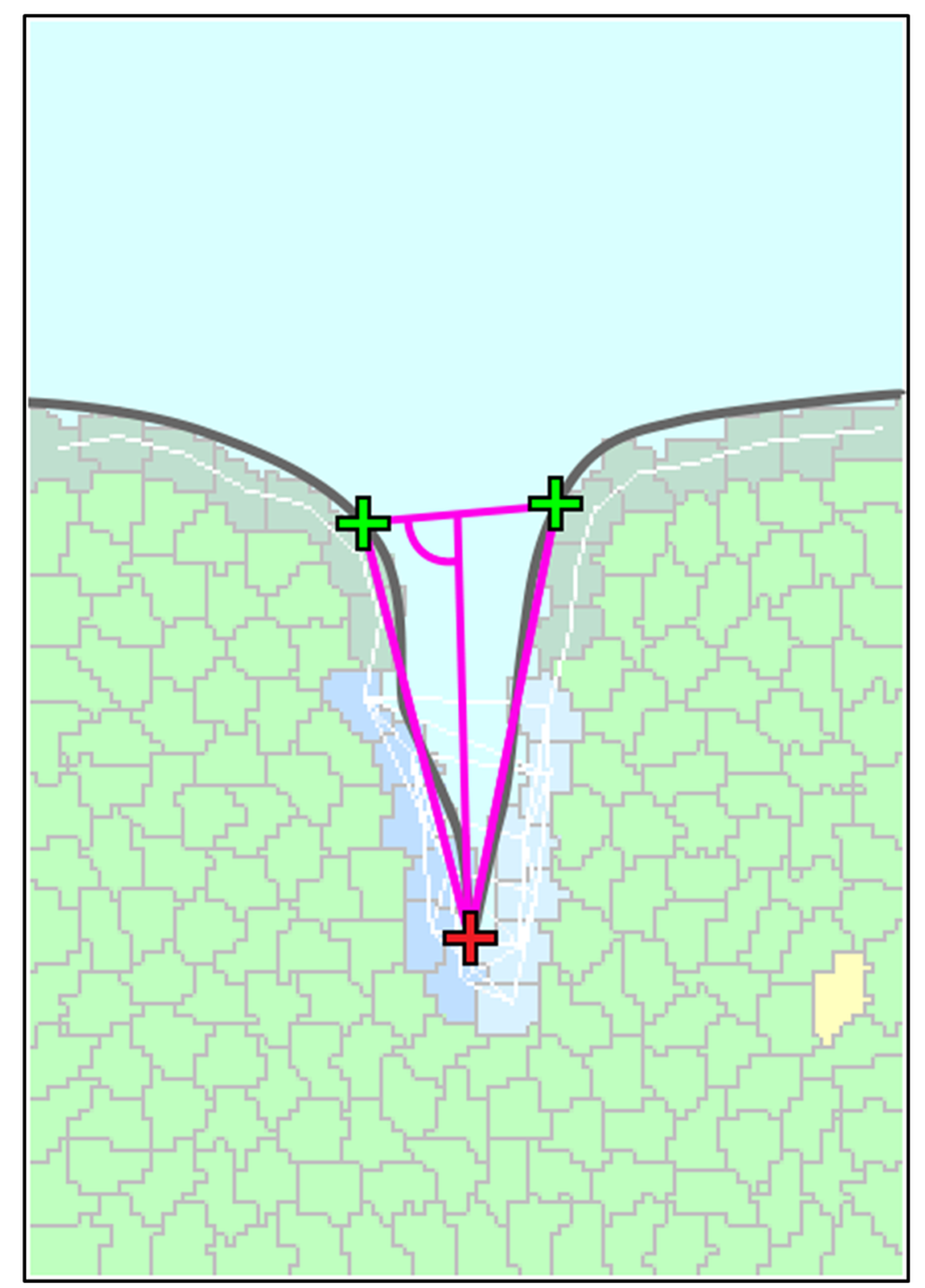

Supplement: Figure S3 — Tilt calculation. The tilt angle was measured as the smaller of the complementary angles formed by the line segment between the extrema, and the line segment from the cleft center to the midpoint. This is used to measure the relative alignment of the clefts to the bud surface. Clefts with a tilt angle of less than 45° were eliminated as “failed clefts”. (TIF) [file pcbi.1003319.s003.tif]

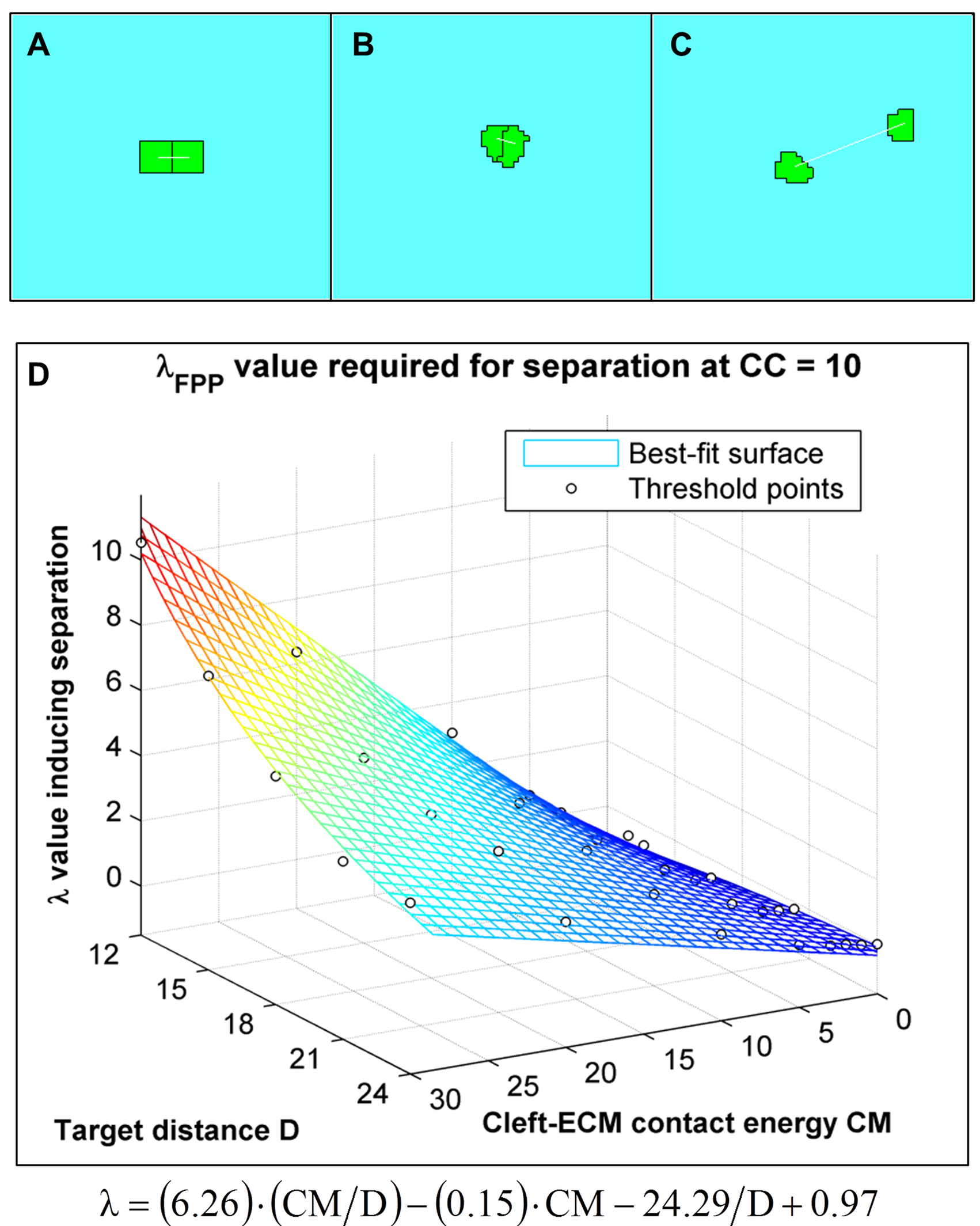

Supplement: Figure S4 — Determination of relationship between focal point plasticity λ value, target distance D, and cleft-ECM contact energy (CM). (a) A simplified simulation was initialized with two 6×6 cells subjected to area and perimeter constraints, (b) A simulation was run for 1000MCS for varying values of D, CM and λ, and final cell distances were recorded. (c) Final stage of cell separation. (d) For each value of D selected, the λ and CM values required to achieve separation were saved and plotted. For these simulations, cell-cell contact energy value (CC) was kept constant at 10. A surface was fitted to these points in the form: This equation approximates the λ value required to achieve separation between two linked and opposing cleft cells under conditions in the single cleft simulation. It was used to select a range of focal point plasticity λ values that allowed us to examine the interplay between cleft-cell adhesion, cell-matrix adhesion, and mitosis rate. (TIF) [file pcbi.1003319.s004.tif]

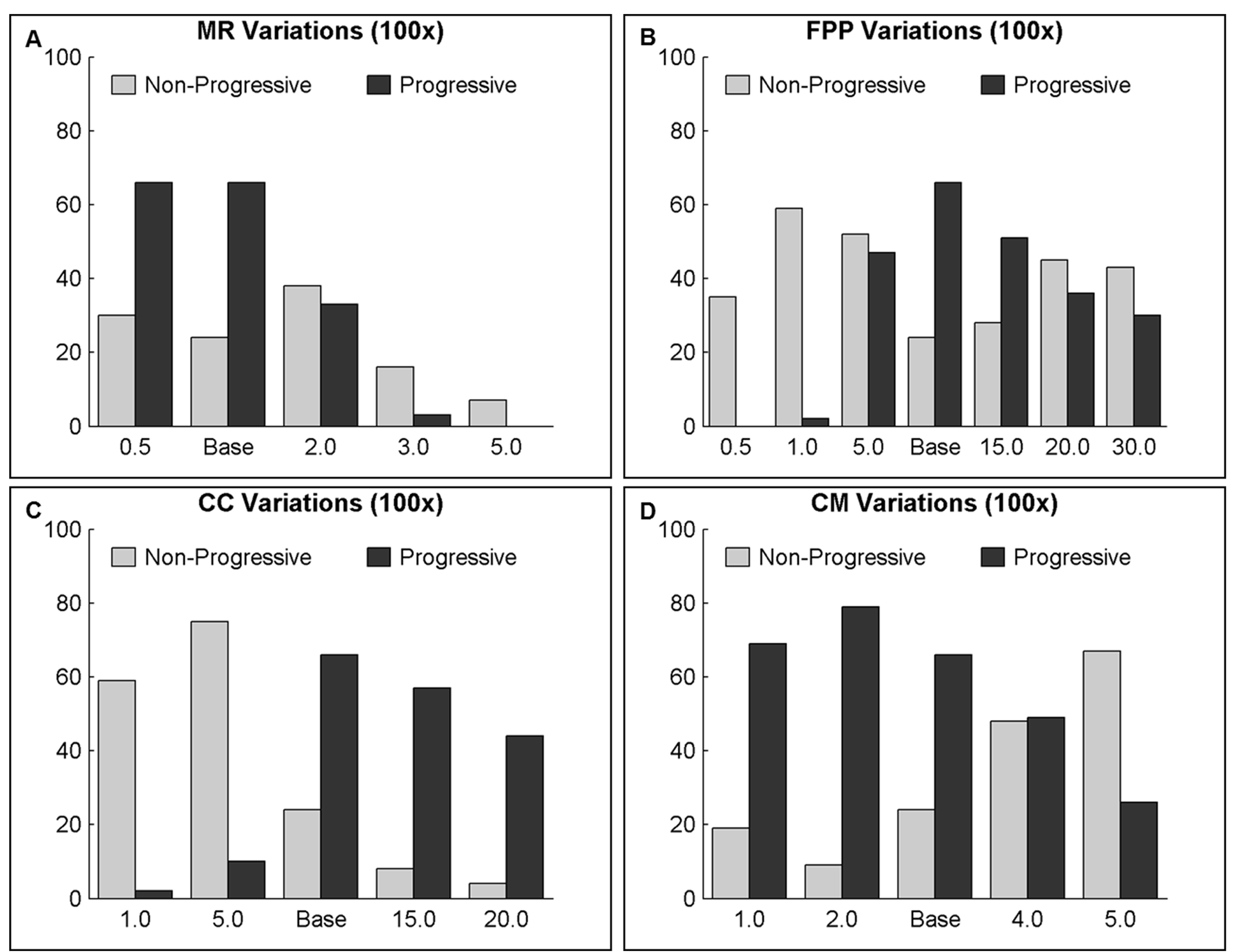

Supplement: Figure S5 — Ratio of progressive to non-progressive clefts obtained during parametric search. (a) Mitotic rate (MR) variation from 0.5% to 5% (b) Focal point plasticity λ (FPP) variations from 0.5 to 30 (c) Cell-cell (CC) contact energy variation from 1 to 20 and (d) Cell-matrix (CM) contact energy variations from 1 to 5. For all the parameters, the corresponding ranges have been chosen based on the number of progressive clefts obtained in comparison to the number of non-progressive clefts and failed clefts. (TIF) [file pcbi.1003319.s005.tif]
