# Supplementary material for: Cell-Based Multi-Parametric Model of Cleft Progression during Submandibular Salivary Gland Branching Morphogenesis
Source: PLoS Comput Biol. 2013 Nov 21;9(11):e1003319. doi: 10.1371/journal.pcbi.1003319 (PMC3836695; doi:10.1371/journal.pcbi.1003319)
Supplement: Table S1 — Cleft categorization during parametric search enabling choice of ranges of values for mitosis rate and focal point plasticity λ. Clefts were categorized as failed, progressive and non-progressive based on cleft depths measured from time-lapse videos of ex-vivo cultured explants. (DOCX) [file pcbi.1003319.s006.docx]

**Table S1** – Cleft categorization during parametric search for mitosis rate and focal point plasticity λ value

| Parameter – Mitosis rate (MR), Focal Point Plasticity (FPP λ) | Number of Failed clefts | Number of Non-progressive clefts | Number of progressive clefts |
| --- | --- | --- | --- |
| MR- 0.5% | 0 | 30 | 66 |
| MR- Base (1%) | 0 | 24 | 66 |
| MR- 2% | 21 | 38 | 33 |
| MR - 3% | 77 | 16 | 3 |
| MR - 5% | 92 | 7 | 0 |
| FPP λ− 0.5 | 65 | 35 | 0 |
| FPP λ− 1 | 39 | 59 | 2 |
| FPP λ− 5 | 1 | 52 | 47 |
| FPP λ− Base (10) | 0 | 24 | 66 |
| FPP λ− 15 | 3 | 28 | 51 |
| FPP λ− 20 | 3 | 45 | 36 |
| FPP λ− 30 | 21 | 43 | 30 |
