# Supplementary material for: Cell-Based Multi-Parametric Model of Cleft Progression during Submandibular Salivary Gland Branching Morphogenesis
Source: PLoS Comput Biol. 2013 Nov 21;9(11):e1003319. doi: 10.1371/journal.pcbi.1003319 (PMC3836695; doi:10.1371/journal.pcbi.1003319)
Supplement: Table S2 — Cleft categorization during parametric search enabling choice of ranges of values for cell-cell and cell matrix contact energies. Clefts were categorized as failed, progressive and non-progressive based on cleft depths measured from time-lapse videos of ex-vivo cultured explants. (DOCX) [file pcbi.1003319.s007.docx]

**Table S2** – Cleft categorization during parametric search for cell-cell and cell matrix contact energies.

| Parameters - Cell-Cell Contact energy ( CC) , Cell-Matrix contact Energy (CM) | Number of Failed clefts | Number of Non-progressive clefts | Number of progressive clefts |
| --- | --- | --- | --- |
| CC - 1 | 39 | 59 | 2 |
| CC - 5 | 15 | 75 | 10 |
| CC - Base (10) | 0 | 24 | 66 |
| CC - 15 | 0 | 8 | 57 |
| CC - 20 | 0 | 4 | 44 |
| CM - 1 | 0 | 19 | 69 |
| CM - 2 | 0 | 9 | 79 |
| CM - Base (3) | 0 | 24 | 66 |
| CM - 4 | 1 | 48 | 49 |
| CM - 5 | 7 | 67 | 26 |
